# A cross-sectional study identifying disparities in serum metabolic profiles among hypertensive patients with ISH, IDH and SDH subtypes

**DOI:** 10.3389/fcvm.2023.1102754

**Published:** 2023-05-04

**Authors:** Yang Shen, Pan Wang, Xinchun Yang, Mulei Chen, Ying Dong, Jing Li

**Affiliations:** ^1^ Department of Nephrology, Beijing Chaoyang Hospital, Capital Medical University, Beijing, China; ^2^ Heart Center & Beijing Key Laboratory of Hypertension, Beijing Chaoyang Hospital, Capital Medical University, Beijing, China; ^3^ Department of Cardiology, Beijing Chaoyang Hospital, Capital Medical University, Beijing, China

**Keywords:** hypertension, isolated diastolic hypertension, combined systolic and diastolic hypertension, metabolomics, isolated systolic hypertension

## Abstract

**Background:**

It has been well acknowledged that disordered intestinal microflora and their fermented products play crucial role during the development of hypertension (HTN). Aberrant profiles of fecal bacteria have been documented in subjects with isolated systolic HTN (ISH) and isolated diastolic HTN (IDH) previously. Nevertheless, evidence regarding the association of metabolic products in the bloodstream with ISH, IDH and combined systolic and diastolic HTN (SDH) remains scarce.

**Methods:**

We performed a cross-sectional study and conducted untargeted liquid chromatography-mass spectrometry (LC/MS) analysis on serum samples of 119 participants, including 13 subjects with normotension (SBP < 120/DBP < 80 mm Hg), 11 individuals with ISH (SBP ≥ 130/DBP < 80 mm Hg), 27 patients with IDH (SBP < 130/DBP ≥ 80 mm Hg), and 68 SDH patients (SBP ≥ 130, DBP ≥ 80 mm Hg).

**Results:**

Here, the results showed clearly separated clusters in PLS-DA and OPLS-DA score plots for patients suffering from ISH, IDH and SDH when compared with normotension controls. The ISH group was characterized by elevated levels of 3,5-tetradecadien carnitine and notable reduction of maleic acid. While IDH patients were enriched with metabolites in L-lactic acid and depleted in citric acid. Stearoylcarnitine was identified to be specifically enriched in SDH group. The differentially abundant metabolites between ISH and controls were involved in tyrosine metabolism pathways, and in biosynthesis of phenylalanine for those between SDH and controls. Potential linkages between the gut microbial and serum metabolic signatures were detected within ISH, IDH and SDH groups. Furthermore, we found the association of discriminatory metabolites with the characteristics of patients.

**Conclusion:**

Our findings demonstrate disparate blood metabolomics signatures across ISH, IDH and SDH, with differentially enriched metabolites and potential functional pathways identified, reveal the underlying microbiome and metabolome network in HTN subtypes, and provide potential targets for disease classification and therapeutic strategy in clinical practice.

## Introduction

Hypertension (HTN) has been widely recognized as a crucial risk factor for multiple cardiovascular diseases (CVD), leading to substantial financial burden globally (1, 2). The prevalence of HTN is rising worldwide, with estimates suggesting a total of 46.4% adult populations suffering from HTN in China (2012–2015) according to the 2017 American College of Cardiology/American Heart Association (ACC/AHA) blood pressure (BP) guidelines (1, 3, 4). Investigators have spared increasing interests and efforts to assess the risks of HTN subtypes including isolated systolic HTN (ISH), isolated diastolic HTN (IDH) and combined systolic and diastolic HTN (SDH). ISH and IDH defined by ACC/AHA guideline were suggested to carry significant risk for left ventricular global longitudinal strain (5). In addition, the contribution of IDH, ISH, and SDH to the incident of heart failure and CVD events was identified to be increased with age (6). Moreover, it was recently demonstrated that different HTN phenotypes were at significant risk of cardiovascular death, and that SDH was associated with higher mortality risk than IDH and ISH (7). Distinct treatment strategies specific for IDH, ISH and SDH are urgently required, however, available literature contains limited data on the pathogenesis and new targets for the early prevention of different HTN subtypes.

Gut microbiota is indicated to be a key factor in the pathogenesis of CVD and is particularly crucial for HTN pathogenesis (8–10). Cumulative researchers have confirmed that patients with hypertensive disease exhibit apparent intestinal microbial dysbiosis, which leads to aberrant high BP (8, 11, 12). Previous studies demonstrate that gut microbes contribute to the pathogenesis of various diseases through their profound impacts on metabolism, and the disordered intestinal microbiota profiles directly result in metabolic alterations and metabolic disorders (13, 14). The small molecule metabolites produced by gut microbes are capable to drive host responses at target organs. In hypertensive patients, significant shifts in microbial metabolic functions and microbiota-derived products known as short chain fatty acids have been documented (11). Meanwhile, by metabolizing dietary phosphatidylcholine, choline, L-carnitine and betaine, gut microbiome has been identified to elicit the accumulation of trimethylamine N-oxide, which is prominently elevated in plasma of hypertensive patients, and facilitates angiotensin II-induced HTN in animal experiments (15). Untargeted metabolomic analyses of serum, plasma and urine specimens from hypertensive patients further revealed apparent variations when compared to normotensive controls (8, 16,17). For distinct HTN subtypes, it has been illustrated that the dysbiosis of gut microbiota differs among HTN, IDH, and ISH patients (18). A link between the variation of fecal bacteria communities and the phenotypes of HTN in ISH and IDH was further elucidated recently (19).

Whereas our knowledge of the complex microbe-metabolism interactions specific to ISH, IDH and SDH remains limited, leaving an understanding gap that greatly delays clinical translation. Thus interest in the metabolic profiles of HTN subtypes is increasing in an attempt to acquire information of how the serum metabolomic signatures specifically correlated with the pathological subtypes manifestation in hypertensive diseases. In light of this, we conducted serum metabolome analysis with liquid chromatography-mass spectrometry (LC/MS) based approaches to elucidate the metabolic signatures of disparate hypertensive phenotypes. The specific serum metabolite profiles and functional pathways associated with ISH, IDH and SDH were identified, respectively. We further explored the relevance of metabolism-microbiota interplay to HTN subtypes.

## Methods

### Study subjects enrollment

We recruited the patients and collected stool and serum samples from our previous study with normotensive controls, pre-hypertensive individuals, and patients diagnosed with HTN (8). Briefly, our present study is a cross-sectional study involving participants from the Kailuan Study from September 2014 to February 2015. The Kailuan study focus on the Kailuan community of Tangshan, a large modern city in northern China, where 11 hospitals in the community responsible for health care, all of which participated in the physical examination. Demographic characteristics of the subjects were recorded, including age, sex, height, weight, body mass index (BMI), fasting blood glucose, total cholesterol and triglyceride. Patients were recruited based on the exclusion criteria as follows: (1) individuals suffered from malignant cancer, stroke, atrial fibrillation, heart failure, renal failure, peripheral artery disease, immunodeficiency disorders, secondary causes of HTN, and patients with previous cardiovascular events; (2) subjects treated with statin, aspirin, insulin, metformin, nifedipine or metoprolol; (3) individuals been exposed to antibiotics, commercial prebiotics or probiotics in the last eight weeks prior to blood collection. All the samples and information were collected when HTN patients were newly diagnosed prior to antihypertensive treatment.

BP of subjects in a sitting position was measured by nurses or physicians under a random-zero mercury column sphygmomanometer. The BP readings were recorded three times at every five minutes intervals. The average reading was calculated and considered to be the final BP data. Healthy controls (Nor) were defined as SBP < 120 mm Hg, or DBP < 80 mm Hg, IDH was diagnosed as SBP < 130 mm Hg and DBP ≥ 80 mm Hg, ISH indicates patients with SBP ≥ 130 mm Hg and DBP < 80 mm Hg, and SDH patients were SBP ≥ 130 and DBP ≥ 80 mm Hg according to the 2017 ACC/AHA guidelines (4, 20). Ultimately 13 controls, 11 ISHs, 27 IDHs and 68 SDHs were proceed to the metabolome examination. Ethics approval was obtained from the Ethics Committee of Beijing Chaoyang Hospital. Signed informed consent have been obtained from all the participants prior to data collection, and this study complied with all applicable institutional regulations regarding the ethical use of information and samples from human participants.

### Sample preparation

The fasting blood samples were harvested from all the participants with coagulation-promoting vacuous tubes. Serum samples were separated by centrifugation at 4°C, 3,000 rpm for 10 min, and stored at −80°C until further examination. Prior to metabolomics determination, each 100 µl serum sample was thawed under room temperature, mixed with 300 µl methanol (80%) and 10 µl DL-o-chlorophenylalanine [2.9 mg/ml, GL Biochem (Shanghai) Ltd.] as internal standard. The mixtures were vortexed and subsequently centrifuged at 4°C, 12,000 rpm for 15 min (8). The supernatant obtained was then subject to untargeted liquid chromatography-mass spectrometry (LC/MS).

### Untargeted metabolome detection based on LC/MS

There were 7 quality control samples and 119 experimental samples for the metabolome detection. LC/MS was performed with hypergod C_18_ column (100 mm × 2.1 mm × 1.9 µm) under the platform (Thermo, Ultimate 3,000LC, Orbitrap Elite). The setting parameters for chromatographic separation was as follows: (1) temperature was maintained at 40°C; (2) the flow rate was at 0.3 ml/min; (3) mobile phase A consisted water with 0.1% formic acid; (4) mobile phase B was acetonitrile with 0.1% formic acid. Each 4 µl sample was injected into the detecting system under 4°C, and then transported through the column by the mobile phase. The mobile phase gradient elution procedure was 95% mobile phase A +5% mobile phase B at 0–2 min, 5% mobile phase A+ 95% mobile phase B at 12–15 min, 95% mobile phase A +5% mobile phase B at 17 min, respectively. And the parameters for ES+ (positive ion mode) detection were heater temperature at 300°C, sheath gas flow rate at 45 arb, aux gas flow rate at 15 arb, sweep gas flow rate at 1 arb; spray voltage on 3.0 kV; capillary temperature at 350°C; S-Lens RF level at 30%. And for ES− (negative ion mode) mode, the ion chromatograms were detected under spray voltage at 3.2 kV and S-Lens RF level of 60%. The total ion chromatogram from each sample was manipulated and metabolic peaks and features were draw using SIEVE software (Thermo). The retention time of each peak was tracked in the total ion current with SIEVE software (Thermo). The data were then normalized and transformed into two-dimensional data matrix with retention time, compound molecular weight, sample peak intensity (21). Only peak area data with <50% null value in the cohort were retained, missing values were recorded with the half minimum value, and each detected metabolic feature was normalized with the total ion current. The peak area was used to normalize the data, with all feature areas of each sample set as 1. The relative abundance of each feature was calculated through standardization. For quality assurance, the relative standard deviation for each peak was calculated, and metabolic features with relative standard deviation of >30% were removed to obtain high quality data set. For metabolite identification, we matched the m/z values and mass of compounds to the featured peaks in the METLIN database, which enhances accurate quantification and facilitates it to more effectively use the data in metabolite databases. The ppm was <20.

### Metabolic functional profiles determination

By matching to the Kyoto Encyclopedia of Genes and Genomes (KEGG) Pathway database (http://www.kegg.jp/kegg/pathway.html), the metabolic features identified to be differentially abundant between groups were found to participate in multiple metabolic pathways (22). Through enrichment analysis and topological analysis of the metabolites-involved pathways, these metabolic pathways were further screened, and the most crucial KEGG pathways correlated with the potential metabolic biomarkers were obtained.

### Gut microbiome annotation

The metagenome shotgun sequencing data of the stool samples assessed in current work were from the EMBL European Nucleotide Archive underthe BioProject accession code PRJEB13870. The metagenomic sequencing of bacterial DNA, gut microbial taxonomic annotation and abundance determination were conducted as we described previously (19). The stool and serum samples were collected from the participants on the same visit.

### Statistical analysis

Demographic characteristics of the subjects were quantitatively represented with mean and SD. Range was described for continuous variables, and count with percent prevalence were shown for categorical variables. *Z*-score as a standard score was dimensionless quantity based on subtracting the mean and then dividing the standard deviation of the sample data. The formula was that *Z*-score = (*x − µ*)/*σ*. *x* indicated a specific score for a specific sample, µ was the mean of the cohort, and σ represented the standard deviation of all the samples (23). For univariate statistical analysis, two-tailed Student's *t*-test was performed to determine whether the two groups of metabolic data were significantly different or not, and *p* < 0.05 was regarded as reaching a statistical significance. Fold change was calculated by the ratio of means between the comparisons. The multivariate statistical analysis method was performed to construct a reliable mathematical model by plotting the clustering or separation of samples from distinct groups using partial least squares discrimination analysis (PLS-DA) and orthogonal partial least squares discriminant analysis (OPLS-DA) models. The serum metabolome data were subjected to multivariate statistical analyses using SIMCA software (V14.1, Sartorius Stedim Data Analytics AB, Umea, Sweden), and samples from HC, IDH, ISH and SDH groups were discriminated into clusters with PLS-DA and OPLS-DA. For sophisticated multivariate statistical modeling in supervised analysis, PLS-DA was applied with regression model and dimension reduction, and the regression results were used for discriminant analysis (24). Furthermore, the non-orthogonal variables and orthogonal variables were used to obtained OPLS-DA score for multi-class classifying the difference between global metabolic profiles between groups, and identify differently altered metabolites (25). Metabolites with *p* < 0.05, and variable importance in the projection (VIP) >1 from the OPLS-DA model, were considered to be statistically different. Volcano plots integrating the fold change and *p*-values were performed to depict the significantly different metabolites. To assess the association of serum metabolites, with clinical measures or gut microbial composition, Spearman's correlation analysis was conducted. The visualizations of correlation heat-maps and networks was conducted with the OmicStudio tools at https://www.omicstudio.cn/tool.

## Results

### Demographic characteristics of the participants

This study included 119 participants from the population based on our previous cohort (8). 13 individuals with normal BP, were compared with 11 patients with ISH, 27 with IDHs and 68 with SDH, according to the ACC/AHA guidelines. The percentages of participants with ISH, IDH and SDH were 9.2, 22.7 and 57.1%, respectively. The detailed baseline characteristics of the study population by normal BP status and HTN subtypes are summarized in Supplementary Table S1. The average age of the participants was 58.5 years, of whom 88.2% were men. The mean age of the participants with ISH was 59 years, and those with IDH or SDH exhibited lower mean age (P (ISH vs. IDH) = 0.016, P (ISH vs. SDH) = 0.030). Compared with individuals with normal BP, those with HTN subtypes showed extremely higher SBP and DBP. Hypertensive patients were more likely to exhibit higher uric acid (except for ISH), and show elevated fasting blood glucose (except for IDH). There was no significant difference in heart rate, BMI, creatinine, total cholesterol, triglyceride, high-density lipoprotein cholesterol, low-density lipoprotein cholesterol, blood platelets and white blood cells between separated groups.

### Heterogeneity of ISH, IDH and SDH individuals in metabolomic profiling

**Figure 1:**
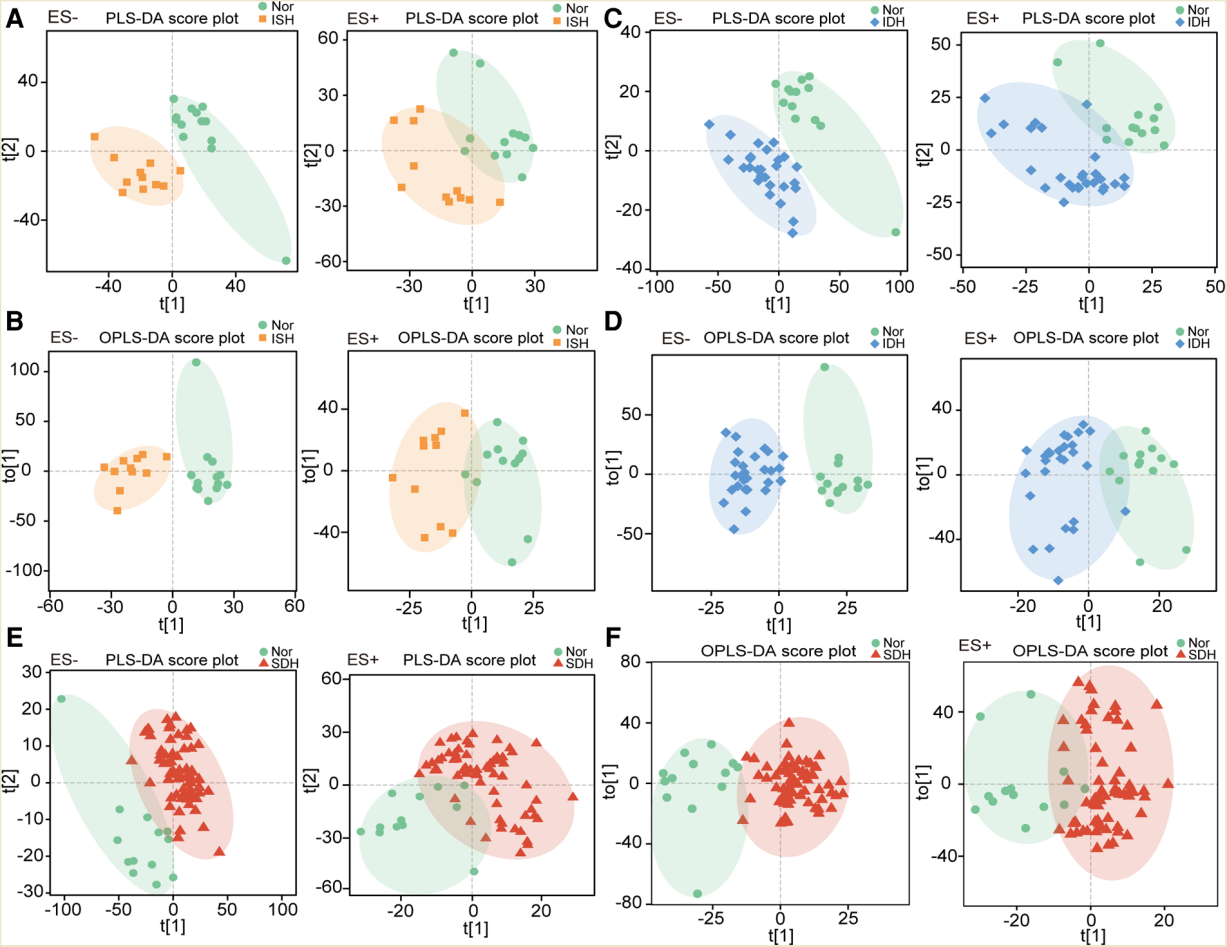
Individuals with ISH, IDH or SDH exhibited dissimilar serum metabolic characteristics as compared with normotensive controls. (**A**) The PLS-DA score plots showing overall metabolic features in ISH patients as compared with controls (Nor) under negative and positive mode, respectively. (**B**) Scatter diagram derived from OPLS-DA illustrated the separation of serum metabolomic data in ISH patients and normotensive controls. Left was in ES− mode, and right was in ES+ mode. (**C**) PLS-DA plots describing the metabolic features in IDH patients as compared with controls. (**D**) OPLS-DA score plots identified the difference in metabolic profiles between IDH patients and Nor with a supervised multiple regression analysis. The results based on data of ES− mode was shown in left, and ES+ mode was in right. (**E**) Metabolic characteristics between SDH and Nor were identified based on PLS-DA plots. F, OPLS-DA showed the separation of serum metabolomic profiles in SDH patients and normotensive controls.

To investigate the specific serum metabolomic features in distinct HTN subtypes, we compared the distributions in PLS-DA and OPLS-DA scatter plots of ISH, IDH and SDH patients with normotensive controls, respectively, by multivariate analysis. Here, we demonstrated that, in both positive and negative ion modes, patients with ISH clustered separately from the healthy controls (Figures 1A,B). As well, IDH patients were significantly deviated in overall metabolomic structure from normotensive controls (Figures 1C,D). PLS-DA and OPLS-DA also identified obvious difference between SDHs and controls (Figures 1E,F). To explore the extent of the similarity of the metabolic signatures between HTN subtypes of ISH, IDH and SDH, we examined the performance in PLS-DA and OPLS-DA between patients with HTN subtypes. Both diagrams displayed apparent departures of ISH and SDH group (Supplementary Figures S1A,B). PLS-DA and OPLS-DA further revealed that IDH patients were separated from SDH participants, although there were overlaps (Supplementary Figures S1C,D). In addition, distinct clusters were confirmed between the ISH and IDH group (Supplementary Figures S1E,F). The aberrant metabolic patterns across ISH, IDH and SDH indicated unique features in distinct HTN subtypes.

### Disparity in metabolic composition across HTN subtypes

**Figure 2:**
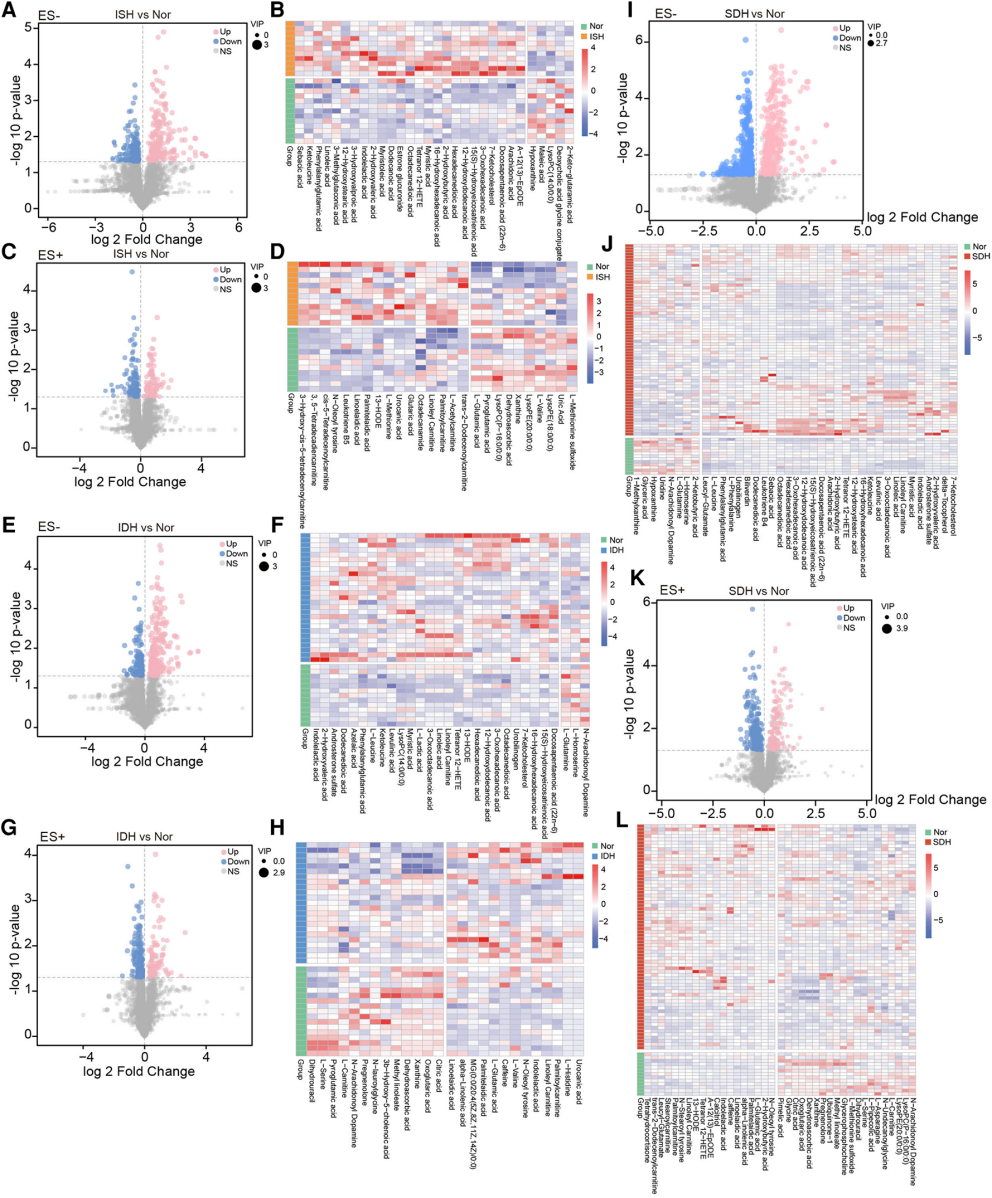
Determination of the dramatically altered metabolites in ISH, IDH or SDH patients as compared with healthy controls. (**A,C**) Volcano plots illustrating the fold changes, *p*-values and VIP of each detected metabolic peaks in ISH and Nor group under negative and positive mode. For significantly varied peaks in ISH, the threshold of VIP in OPLS-DA model was >1, and the relative abundance was additionally screened with *p*-values <0.05 from two-tailed Student's *t*-test. Dots represented metabolites, blue denoted down-regulated, pink represented those up-regulated in ISH, and grey were not significant different peaks. Dot size showed the value of VIP. (**B,D**) Relative abundance of each metabolite identified to be prominently altered in ISH was described in heatmaps. Only those successfully identified were depicted. **B** shows those detected in ES-, and **D** displays those in ES+. (**E,G**) Metabolites shifted in IDH patients were detected with volcano plots. E was derived based on metabolomics data in ES− mode, and G was in ES+ mode. Dots denoted metabolites detected, blue ones were those reduced in IDH, and pink represented those enriched in IDH. (**F,H**) Among the markedly varied metabolites between IDH and Nor group, the relative abundance of those successfully identified were further shown in hierarchical cluster analysis heat-maps. **F** for those in ES-, and H for those in ES+. (**I,K**) Volcano plots identifying the discriminative metabolites between SDH and Nor was conducted with the OPLS-DA model. I was based on metabolomics data in ES− mode, and K was in ES+ mode. The dots represented various metabolites, and color denoted significantly down-regulated or up-regulated in SDH. (**J,L**) Of the varied metabolites between SDH and Nor, heatmaps illustrated comparison for the relative abundance of those identified metabolites with significant disparate levels.

**Figure 3:**
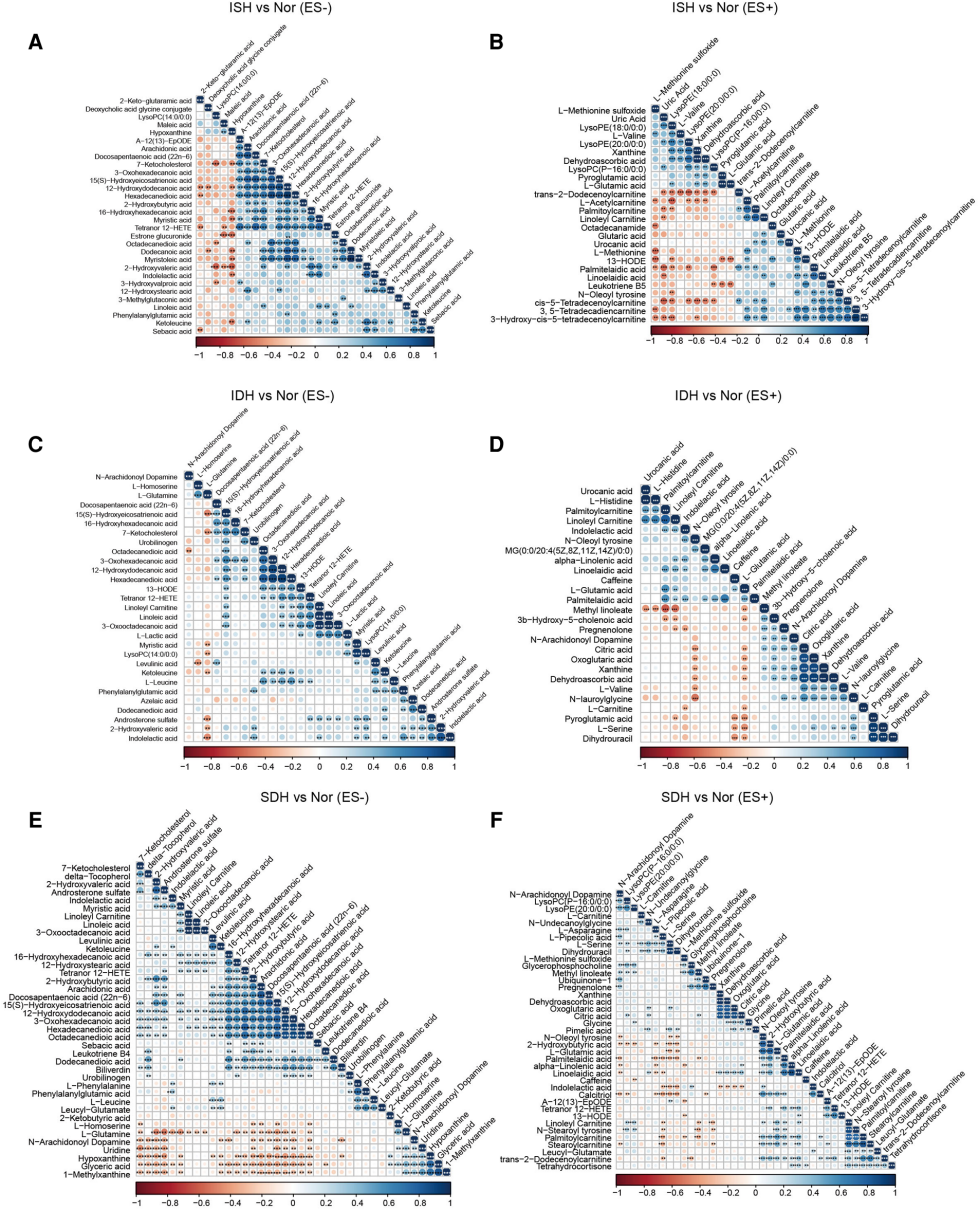
Association analysis of the metabolites disparate among groups by Spearman correlation. (**A,B**) Co-abundance correlation of the distinct metabolites identified in ISH patients as compared with normotensive controls under ES− and ES+ mode, respectively. (**C,D**) Variants with significant different metabolic compositions between IDH and Nor in negative and positive mode were shown to be related with each other. (**E,F**) Heat map depicting the potential correlation of differently abundant metabolites identified between SDH and controls. (**E**) For those in ES−, and (**F**) for ES+. Negative correlation was depicted in orange and positive correlation was in blue. The shade of and size of dots represented the correlation coefficient. *p < 0.05, **p < 0.01, ***p < 0.001; derived from Spearman's correlation.

Next, we focused on serum metabolome variations that significantly changed in each hypertensive disease status. Volcano plots revealed 411 and 220 differentially enriched metabolites (DEMs) between the ISH and control group under negative and positive ion mode, respectively (Figures 2A,C). Of these discriminatory metabolic products, a total of 56 metabolites were accurately identified, as described in Figures 2B,D. The ISH group was characterized by significantly higher levels of 3,5-tetradecadien carnitine, arachidonic acid, hexadecanedioic acid, 7-ketocholesterol, linoleic acid, linoleyl carnitine and glutaric acid, but lower hypoxanthine, maleic acid, l-glutamic acid levels (Figures 2B,D). Volcano plots with 386 (ES-) and 255 (ES+) metabolites at *p* < 0.05 and VIP > 1 from the OPLS-DA model, showed the specific DEMs involved with IDH (Figures 2E,G). Heatmap distribution diagrams showed clear alterations in the metabolites characterized by higher 16-hydroxyhexadecanoic acid, octadecanedioic acid, linoleyl carnitine, L-lactic acid, linoelaidic acid and palmitelaidic acid levels in the IDH group (Figures 2F,H). However, N-arachidonoyl dopamine, L-glutamine, citric acid and pyroglutamic acid levels were significantly decreased in the IDH patients. To examine the metabolic biomarkers that could distinguish SDH from controls, we performed comparison of metabolic profiles between these groups. 625 and 355 metabolic features in ES− and ES+ were considered as DEMs in SDH (Figures 2I,K). The DEMs linoleic acid, 15(S)-hydroxyeicosatrienoic acid, linoleyl carnitine and stearoylcarnitine were more abundant in the SDH group, while 2-ketobutyric acid, hypoxanthine, glyceric acid, N-arachidonoyl dopamine and dehydroascorbic acid were more abundant in the control group (Figures 2J,L). The complicated co-abundance relationship among these key DEMs was shown in Figure 3, which was extremely strong among the distinct metabolites identified in SDH.

Also, in volcano plots across HTN subtypes, they exhibited dramatic variations in relative abundance of specific metabolites, which revealed that the metabolic patterns were unique in distinct hypertensive patients (Supplementary Figure S2A,C,E,G,I,K). A total of 30 significantly changed metabolites between the ISH group and SDH group samples were identified, as illuminated in the heat map, of which, 9 showed a reduced trend, and 21 displayed an increasing trend in SDH (Supplementary Figures S2B,D). Briefly, indolelactic acid, phenylacetylglutamine, N-stearoyl tyrosine and palmitoleoylethanolamde exhibited lower concentrations in ISH patients than in SDHs. The heat map in Supplementary Figure S2F,H revealed that IDH patients had a different metabolic pattern from SDHs based on these 18 DEMs. In particular, the levels of diaminopimelic acid, hydroxykynurenine, deoxycholic acid and L-acetylcarnitine were significantly increased in SDH patients, whereas 2-hydroxybutyric acid, biliverdin and bilirubin were evidently reduced. Notably, the disparate endogenous metabolites in serum samples were basically related to myristoleic acid, myristoleic acid, 12-hydroxydodecanoic acid, hexadecanedioic acid, maleic acid, stearidonic acid and taurochenodeoxycholic acid when comparison was performed between IDH and ISH group (Supplementary Figures S2J,L). Supplementary Figure S3 described the correlation of various DEMs in distinct HTN patients.

Multivariate linear regression analysis for distinct metabolites obtained among hypertension subtypes was conducted to adjust the major biological confounders. The independent strength of the associations between hypertension subtypes and the top 5 most disparate metabolites identified under positive and negative modes between groups was evaluated. ISH was associated with higher relative abundance of hexadecanedioic acid, 7-ketocholesterol, 13-HODE and palmitelaidic acid independent of the effects of sex, FBG and hemoglobin (Supplementary Tables S2, S3, *P*_hexadecanedioic acid _= 0.037, *P*_7-Ketocholesterol _= 0.002, *P*_13-HODE _= 0.029, *P*_palmitelaidic acid _= 0.013). IDH predicted increases in the relative abundance of levulinic acid, hexadecanedioic acid and L-glutamic acid independent of the effects of sex and uric acid (Supplementary Tables S4, S5, *P*_levulinic acid _= 0.034, *P*_hexadecanedioic acid _= 0.004, *P*_L-glutamic acid _= 0.008). The independent strength of the associations between SDH and top 5 altered metabolites was weakened by adjusting the effects of sex, uric acid, FBG and hemoglobin (Supplementary Tables S6, S7). For different metabolites between ISH and IDH, IDH predicted increases in the relative abundance of L-homoserine, estrone glucuronide and octadecanamide independent of the effects of age and FBG (Supplementary Tables S8, S9, *P*_L-homoserine _= 0.005, *P*_estrone glucuronide _= 0.002, *P*_octadecanamide _= 0.026). Among the distinct metabolites between ISH and SDH, SDH was associated with the relative abundance of L-homoserine, deoxycholic acid glycine conjugate and ornithine independent of the effects of age (Supplementary Tables S10, S11, *P*_L-homoserine _= 0.028, *P*_deoxycholic acid glycine conjugate _= 0.045, *P*_ornithine _< 0.001).

### Functional pathway alterations identified via KEGG enrichment analysis

**Figure 4:**
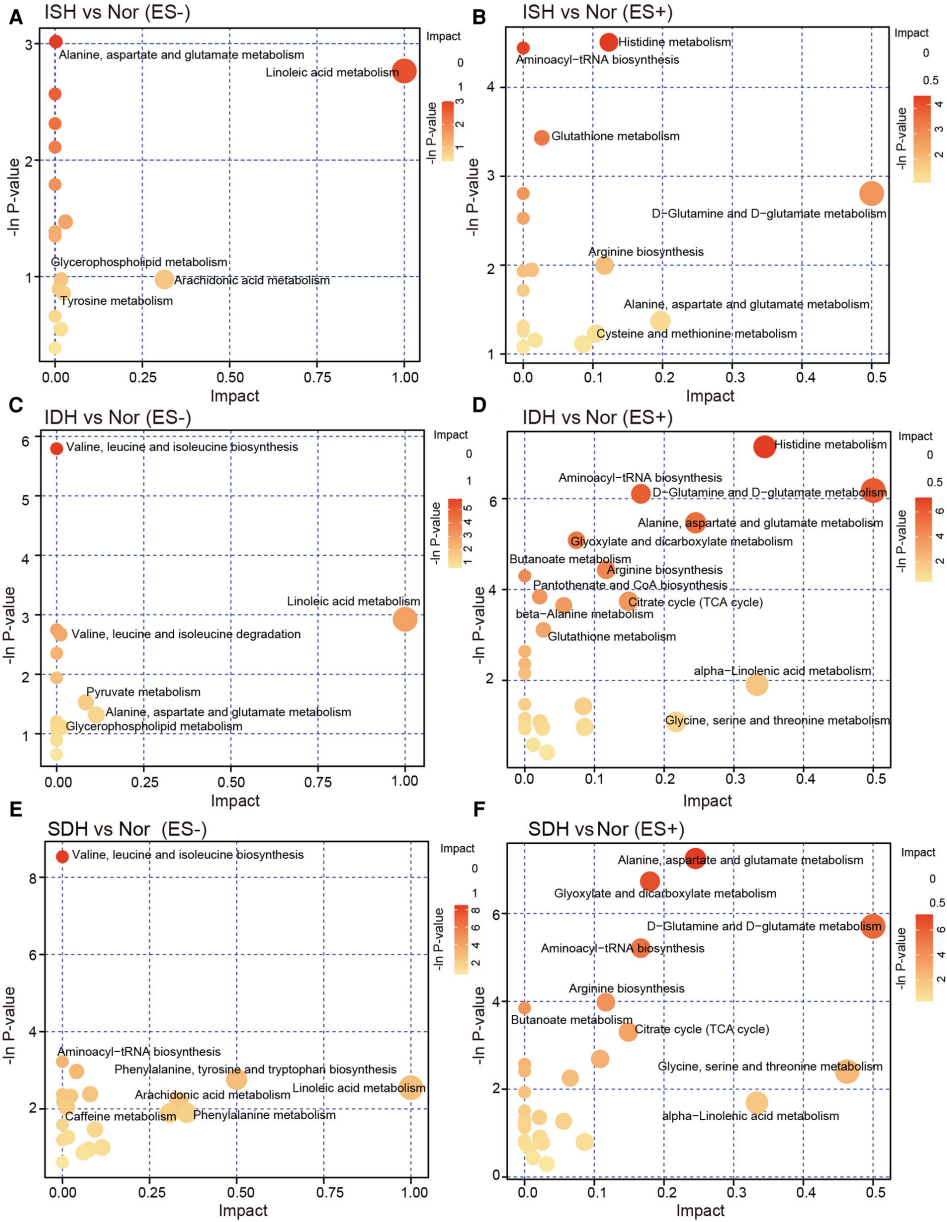
KEGG pathway analysis of the differentially enriched metabolites in ISH, IDH and SDH patients, respectively. (**A,B**) Bubble plots in negative and positive mode exhibited the enriched metabolic pathways of altered metabolic compounds between ISH and healthy controls. (**C,D**) Metabolites significant distinct between IDH group and controls were annotated to KEGG pathways, and the enrichment of metabolite pathways were described. (**E,F**) The significant matched KEGG pathway terms based on altered metabolites between patients with SDH and Nor were shown in the plots. The dots were colored with the −ln *p*-value, and where the color was darker, the enrichment degree was more significant. The dots were further sized according to value of impact factor in each pathway.

The identified DEMs were subsequently mapped to metabolic pathways according to KEGG database, including metabolism of alanine, aspartate and glutamate, linoleic acid, D-glutamine and D-glutamate, and biosynthesis of aminoacyl-tRNA and arginine, which were consistent in each HTN subtype when compared with healthy controls (Figures 4A–F). We noted that pathways involved in tyrosine metabolism was exclusively between ISH and control (Figures 4A,B). And KEGG pathway terms, such as biosynthesis of phenylalanine, tyrosine and tryptophan, and metabolism of phenylalanine were specifically detected in SDH vs. control (Figures 4E,F). Furthermore, across each HTN subtypes, it was observed that DEMs between ISH and SDH mainly functioned in pyruvate metabolism and arginine biosynthesis, whereas those between IDH and SDH primarily participated in porphyrin, pyrimidine metabolism and arginine biosynthesis (Supplementary Figures S4A–D). According to DEMs and differential KEGG metabolic pathways noted between ISH and IDH, the metabolism of pyruvate, tyrosine and linoleic acid was the most significant, which suggested that a disturbance of these pathways might occur in patients (Supplementary Figures S4E,F). The evidence seems to suggest that ISH, IDH and SDH patients may experience aberrant metabolic processing but distinct functional activities.

### Correlation between discrepant metabolites and gut microbiome

**Figure 5:**
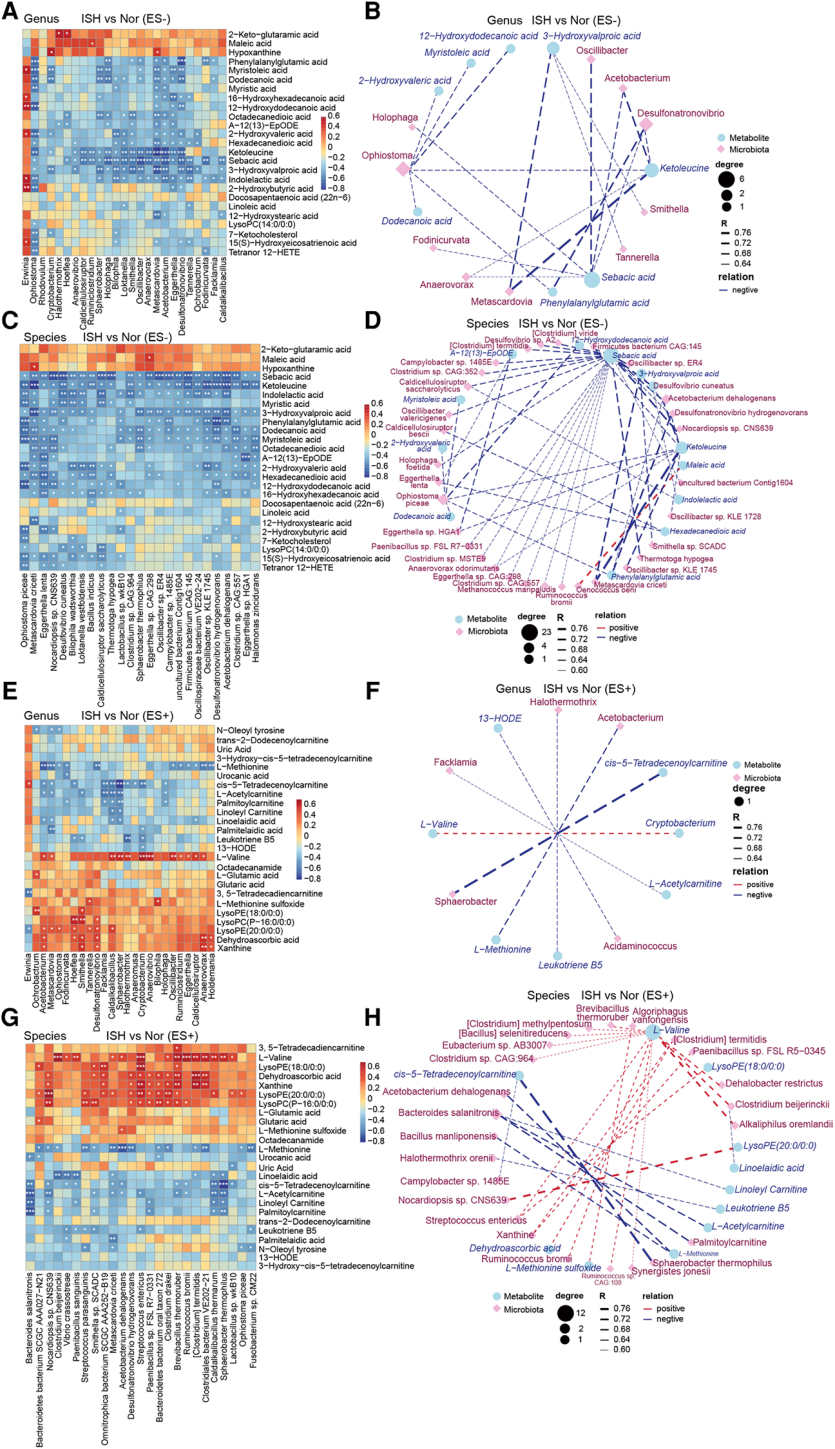
ISH-associated metabolites were identified to be correlated with intestinal microbiota distinguish ISH vs. control individuals. (**A,C,E,G**) The correlations of gut microbial genera and species with the top25 varied serum metabolites in ISH were shown with heat-map. The genera/species examined were those significantly disparate between ISH and controls. A and E depicted the correlation between gut genera and serum metabolites in ES− and ES+, while C and G were the association of microbial species and metabolites. Positive association was in red color, and negative association was in blue. *p < 0.05, **p < 0.01, ***p < 0.001, Spearman's rank correlation. (**B,D,F,H**) Network exhibiting the correlation of metabolome and microbiome at genus and species levels, respectively. Differential microbial genera/species were closely linked to differential metabolites in ES− and ES+ between ISH and control. (**B**) Metabolite in ES− correlated with genera; (**D**) metabolite in ES− correlated with species; (**F**) metabolite in ES+ correlated with genera; (**H**) metabolite in ES+ correlated with species. Blue circle denoted metabolites, and purple rhombus represented bacterial genera/species. Size of circles or rhombus denoted the degree according to the number of variances connected. The correlation coefficient was ≥0.6 or ≤−0.6, and *p*-value was <0.05, as calculated with Spearman correlation. The thickness of lines connecting variants represented the correlation coefficient.

The relative abundance of specific genera and species markedly altered between groups was obtained through taxonomic analysis based on metagenomic sequencing data from our previous work (8, 24). Correlation matrixes were created using Spearman correlation and co-abundance network analysis to explore the potential relationships between changes in the metabolic products and gut microbiome was performed (Figure 5, Supplementary Figures S5, S6).

Between the ISH and control groups, we found that most of ISH-related serum metabolites were significantly correlated with at least one individual connection. In particular, the levels of ketoleucine, sebacic acid, 3-hydroxyvalproic acid and indolelactic acid detected in ES− mode, were negatively correlated with the abundance of genus *Sphaerobacter*, *Bilophila*, *Loktanella*, *Oscillibacter*, *Metascardovia*, *Acetobacterium* and *Desulfonatronovibrio*, and species *Oscillibacter sp.*, *Desulfonatronovibrio hydrogenovorans*, *Acetobacterium dehalogenans*, and *Metascardovia criceti* (Figures 5A,C). Co-occurrence network further showed the complicated connections between intestinal bacteria and DEMs in ISH and controls (Figures 5B,D). Sebacic acid and ketoleucine were identified to be crucial DEMs, which negatively linked to most gut bacteria examined. For ES+ mode, it was found that L-methionine was negatively correlated with the abundance of multiple bacteria such as *Acetobacterium*, *Metascardovia*, *Oscillibacter*, *Ruminiclostridium*, *Eggerthella*, *Caldicellulosiruptor* and *Clostridium sp.*, whereas L-valine was positively related to them (Figures 5E–H).

We also correlated the relative abundance of individual genus and species shifted in IDH, with the DEMs to find key metabolites that were closely associated with abnormalities occurred in IDH (Supplementary Figure S5). Vital DEMs in ES− including 2-hydroxyvaleric acid, hexadecanedioic acid, dodecanedioic acid, ketoleucine and levulinic acid identified between IDH and controls, were observed to be positive to *Alicycliphilu*s and *Alicycliphilus denitrificans*, while negative to *Syntrophobotulus glycolicus*, *Desulfonatronovibrio hydrogenovorans*, *Tuberibacillu*s *calidus*, *Clostridium sp.*, *Oscillibacter sp.*, *Roseburia hominis* (Supplementary Figures S5A–D). In addition, indolelactic acid was negatively associated with the majority of gut microbes (Supplementary Figures S5E–H).

Correlation analysis of serum metabolites varied in SDH, with key fecal microﬂora biomarkers revealed that ketoleucine, urobilinogen, 7-ketocholesterol, myristic acid and 12-hydroxystearic acid were the most significant microbiome-relevant DEMs in negative ion mode (Supplementary Figures S6A–D). Especially, urobilinogen was negatively related with *Acetobacterium dehalogenans*, *Anaerotruncus colihominis*, *Oscillibacter sp.*, *Clostridium sp.*, *Roseburia hominis* and *Firmicutes bacterium*. Whereas 12-hydroxystearic acid showed positive correlation with *Klebsiella sp.* such as *Klebsiella pneumoniae*, which has been confirmed to drive high BP and HTN in mice (26). Supplementary Figures S6E–H described the relationships of gut genera/species and metabolites in ES+ between SDH and controls. *Clostridium sp.*, *Roseburia hominis*, *Firmicutes bacterium* and *Robinsoniella sp.* were positively connected with L-serine, L-pipecolic acid and citric acid, but negatively with calcitriol and N-oleoyl tyrosine (Supplementary Figures S6E–H). Our findings have drawn tight lines between specific metabolites in serum and dysregulation of intestinal microbiome in HTN subtypes.

### Serum metabolic associations with clinical characteristics

**Figure 6:**
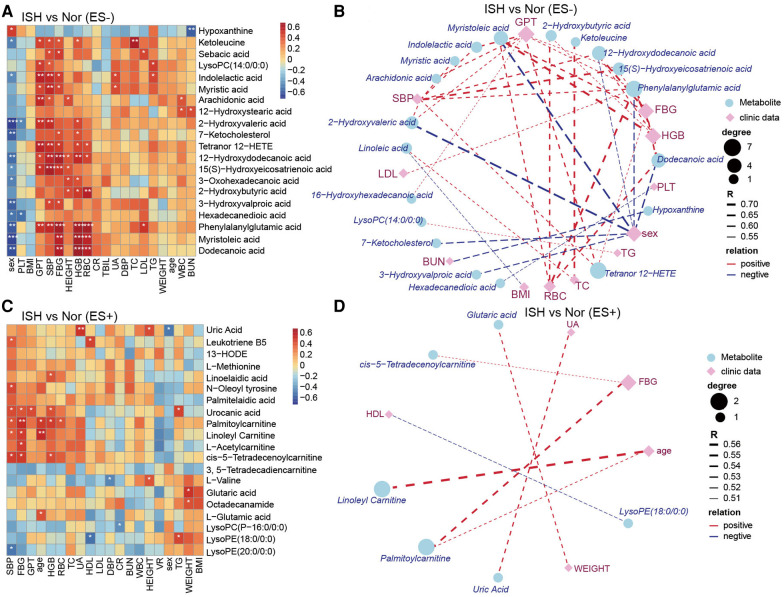
The relevance of clinical parameters with the serum metabolites varied specifically in ISH. (**A,C**) Heat-map depicting Spearman's rank correlation of the top20 differential metabolic compounds between groups and all the clinical indexes of individuals. A for ES− and C for ES+. Red indicated positive association; blue represented negative association. The statistical significance was expressed with *p < 0.05, **p < 0.01, and ***p < 0.001, respectively. (**B,D**) Association networks showed correlations between clinical data and metabolites distinct in ISH vs. Nor. The correlation coefficient was ≥0.5 or ≤−0.5, and *p*-values were <0.05, derived from Spearman correlation analysis. Blue circles denoted metabolites, and purple rhombus represented clinical parameters. Circle or rhombus size represented degree. Red lines were positive correlation, while blue lines indicated negative association. Lines thickness was based on the correlation coefficient.

As considerable variability was observed in the metabolome across the samples in each group, we further explore the linkage of these discriminatory metabolites and the characteristics of patients with HTN subtypes. Our results revealed that ISH-related serum DEMs, including 12-hydroxydodecanoic acid, phenylalanylglutamic acid and palmitoylcarnitine, were positively related to SBP, FBG, HGB and RBC but negatively to sex of the participants (Figures 6A–D). Another noteworthy finding was the DEMs obtained when comparison performed between IDHs and normotension controls, were predominantly associated with the DBP, GPT, HGB and RBC (Supplementary Figures S7A–D). For example, indolelactic acid, 2-hydroxyvaleric acid, 12-hydroxydodecanoic acid, phenylalanylglutamic acid and L-leucine were tightly linked to these clinical characteristics. Likewise, Spearman's correlation was used to assess the pairwise correlations between clinical measures and SDH-related serum metabolites (Supplementary Figure S8). Specifically, DEMs that were significantly associated with both SBP and DBP, such as biliverdin, myristic acid, arachidonic acid, hexadecanedioic acid, 16-hydroxyhexadecanoic acid, tetranor 12-HETE, L-asparagine, L-serine and L-pipecolic acid were also related to GPT (Supplementary Figures S8A–D). Overall, the network between clinical performance and DEMs was more complicated in ISH and IDH, whereas concise and weaker in SDH. These observations implied that the intestinal flora affected HTN subtypes possibly by affecting the metabolism of certain metabolites, which might, in turn, functioned on BP and even GPT, FBG, HGB and RBC levels of the host.

## Discussion

In this study, we present the first analysis of serum metabolome across ISH, IDH and SDH patients to improve our previous knowledge regarding HTN, microbiome and metabolism. It is uncovered that the serum metabolic characteristics, as assessed by PLS-DA and OPLS-DA distributions of ISH, IDH and SDH patients, are significantly distinct from that of normotensive subjects. Comparison for metabolome composition of serum samples in HTN subtypes reveal 57, 55 and 78 DEMs between normotension and ISH, IDH and SDH respectively. The parallel assessment of ISH, IDH and SDH made it possible to identify shared and specific metabolic associations in HTN subtypes.

On the one hand, among these differential metabolites, various metabolites such as Tetranor 12-HETE, 13-HODE and 12-hydroxydodecanoic acid are enriched simultaneously in ISH, IDH and SDH group. On the other hand, several metabolites are abundant specifically in a single HTN subtype. For instance, acylcarnitines (ACs) including 3,5-tetradecadien carnitine, cis-5-tetradecenoylcarnitine are uniquely increased in ISH patients, L-lactic acid is particularly in IDH group, while stearoylcarnitine is inimitably in SDH patients. In addition, the identified DEMs are annotated to metabolic pathways in terms of KEGG database, including alanine, aspartate and glutamate metabolism, D-glutamine and D-glutamate metabolism as well as aminoacyl-tRNA biosynthesis, which is consistent for each HTN subtype when compared to controls.

Major overlapping differences observed between ISH, IDH and SDH when compared with controls emphasize the enrichment of Tetranor 12-HETE. Tetranor 12-HETE as a kind of oxylipin with pro-inflammatory properties, has been documented to participate in the inflammatory response and accelerate the progression of HTN (27). Previously, it was reported that the abundance of Tetranor 12-HETE was promoted by high-dose cadmium intervention, which elicited nephrotoxicity and oxidative stress to the kidneys (28). Meanwhile, Li Q and colleagues demonstrated that plasma level of Tetranor 12-HETE was extremely elevated in patients suffering from nonalcoholic fatty liver disease (29). Furthermore, another study confirmed the significant association between Tetranor 12-HETE and liver fibrosis in nonalcoholic steatohepatitis patients (30). Here in our present work, when examining the serum metabolome in HTN, we consistently observed apparent augment in the content of Tetranor 12-HETE among ISH, IDH and SDH patients in comparison with healthy controls. In addition, we revealed that the presence of Tetranor 12-HETE exhibited a significantly positive correlation with both SBP and DBP levels. Subsequently, it was further identified that Tetranor 12-HETE was negatively associated with *Eggerthella lenta* and *Roseburia hominis* in ISH and SDH patients, respectively. Notably, *Roseburia hominis* has been suggested as a biomarker of HTN, and is considered to be major producer for short chain fatty acids exhibiting protective properties against hypertensive disease (31, 32). Thus our data open the possibility that Tetranor 12-HETE might be an important mediator playing crucial role during the crosstalk between intestinal microbial environment and HTN development.

By considering each HTN subtype in parallel, we also observed multiple metabolites that are typically overproduced in disparate hypertensive disorders. Specifically, ACs including 3,5-tetradecadien carnitine, cis-5-tetradecenoylcarnitine significantly increased among ISH patients. ACs, are intermediate oxidative metabolites that comprise a fatty acid esterified to a carnitine molecule (33, 34). They are produced by mitochondrial and peroxisomal enzymes, including the carnitine palmitoyltransferase (CPT) 1 and 2 enzymes, in order to transport long-chain fatty acids across the mitochondrial membrane for β-oxidation (35). A recent study involving plasma metabolomics profile of overweight individuals with a high visceral fat area showed higher plasma levels of medium- and long-chain ACs as compared to individuals with low visceral fat area (36). Our observation is also in line with previous evidence indicating that pulmonary HTN was strongly associated with higher concentrations of long-chain ACs (37).

Lactic acid (L-lactic acid) is a dead-end product of glycolysis, which plays an essential regulatory role in a variety pathophysiological processes. Previously, the anti-inflammatory effect of lactic acid has been subtly demonstrated by Hoque et al. in a study showing that transient lactic acid exposure suppresses inflammation and alleviates tissue damages in chemically induced pancreatitis and hepatitis models (38). Moreover, lactic acid derived from glycolysis in astrocytes could be transported via monocarboxylate transporters to neurons, where it exerts signaling functions and stimulates the expression of protective genes involved in long-term memory formation (39). However, in contrast to these findings, lactic acid is also suggested as a signaling molecule and regulator participating in multiple diseases, such as ischemic tissue injury, and cancer progression (39). Still, GPR81-mediated signaling activated by lactic acid has been shown to exacerbate ischemic brain injury in the oxygen-glucose deprivation and middle cerebral artery occlusion models (40). When considering cancer growth, Walenta et al. have identified prominently abundant lactic acid accumulation surrounding the tumor tissues, which is significantly higher than that in normal tissue (41). And lactic acidosis in cancer patients is frequently correlated with rapid cancer growth, metastasis and poor survival (39). In the current work, our data provides information that the enrichment of lactic acid was more pronounced in IDH patients, but not ISH or SDH. It is therefore an open question whether lactic acid observed in IDH may contribute to health or illness.

Homoplastically, SDH patients displayed exclusive metabolic alterations from IDHs and ISHs when compared with controls, especial for the expansion of stearoylcarnitine. Stearoylcarnitine is an important long-chain ACs, which has been previously confirmed to be elevated in the blood of obese individuals and accumulate in β cells of subjects with type 2 diabetes, leading to insulin arrest and energy deficiency in key energy pathways (42). Within hypoxia-ischemic brain injury animal models, Dave and colleagues have demonstrated that the level of stearoylcarnitine was primarily increased in the cortex, striatum/thalamus as well as cerebellum (43). Moreover, in pulmonary arterial HTN animal models, another study indicated that stearoylcarnitine was slightly elevated compared with healthy controls, with marginally significant (44). These observations perhaps suggested a causal influence of stearoylcarnitine on mentioned disease, but there is limited knowledge of whether stearoylcarnitine contribute directly to SDH disease, and further attempts to clarify this should be a priority.

Nevertheless, there are several limitations regarding this cross-sectional study. First, the relatively small sample size of the participants is a general challenge for metabolome studies, which might limit the power of the analysis, although we did detect certain statistically significant compounds. Second, the shifts of metabolites composition in patients having acquired ISH, IDH or SDH could not be elucidated as the cause of the disease or a secondary phenomenon following disease onset according to the current findings. Delineation of the causality relationship between gut microbiome, metabolic alterations and disease development should be prioritized in further studies focusing on HTN. Additionally, non-targeted LC-MS analyses were performed to evaluate the metabolite composition in hypertensive phenotypes of ISH, IDH and SDH. Target mass spectrometry is a more sensitive and quantitative method, which would allow further confirmation of the metabolic features observed in the current work.

## Conclusion

In summary, we highlight a distinctive metabolome-based characteristic for ISH, IDH, SDH and normotension. Metabolites as represented with Tetranor 12-HETE and 12-hydroxydodecanoic acid are enriched simultaneously in ISH, IDH and SDH patients. Whereas other metabolites like 3,5-tetradecadien carnitine and cis-5-tetradecenoylcarnitine are particularly elevated in ISH patients, L-lactic acid in IDH subjects, while stearoylcarnitine in SDH patients. These DEMs exert functional role in alanine, aspartate and glutamate metabolism, D-glutamine and D-glutamate metabolism. Our findings provide insights into metabolome disturbances that may play an essential role in linking gut microbial environment and the pathological subtypes manifestation of HTN. This study also suggests potentially metabolic targets for disease classification and therapy to mitigate the damages on cardiometabolic health. Targeting disparate metabolites and gut microbes may provide strategies for the prevention and treatment of distinct subtypes of HTN specifically.

## Data Availability

The original contributions presented in the study are included in the article/**Supplementary Material**, further inquiries can be directed to the corresponding authors.

## References

[1] Mills KT Stefanescu A He J. The global epidemiology of hypertension. Nat Rev Nephrol. (2020) 16:223–37. 10.1038/s41581-019-0244-2 32024986 PMC7998524

[2] Zhou B Perel P Mensah GA Ezzati M. Global epidemiology, health burden and effective interventions for elevated blood pressure and hypertension. Nat Rev Cardiol. (2021) 18:785–802. 10.1038/s41569-021-00559-8 34050340 PMC8162166

[3] Wang Z Chen Z Zhang L Wang X Hao G Zhang Z Status of hypertension in China: results from the China hypertension survey, 2012–2015. Circulation. (2018) 137:2344–56. 10.1161/CIRCULATIONAHA.117.032380 29449338

[4] Whelton PK Carey RM Aronow WS Casey DE Jr Collins KJ Dennison Himmelfarb C 2017 ACC/AHA/AAPA/ABC/ACPM/AGS/APhA/ASH/ASPC/NMA/PCNA guideline for the prevention, detection, evaluation, and management of high blood pressure in adults: a report of the American college of cardiology/American heart association task force on clinical practice guidelines. J Am Coll Cardiol. (2018) 71:e127–e248. 10.1016/j.jacc.2017.11.006 29146535

[5] Nakanishi K Daimon M Yoshida Y Ishiwata J Sawada N Hirokawa M Blood pressure categorization and subclinical left ventricular dysfunction in antihypertensive medication-naive subjects. ESC Heart Fail. (2022) 9:1766–74. 10.1002/ehf2.13860 35199967 PMC9065812

[6] Suzuki Y Kaneko H Yano Y Okada A Itoh H Matsuoka S Age-dependent relationship of hypertension subtypes with incident heart failure. J Am Heart Assoc. (2022) 11:e025406. 10.1161/JAHA.121.025406 35475350 PMC9238621

[7] Bo Y Yu T Guo C Chang LY Huang J Wong MCS Isolated systolic or diastolic hypertension and mortality risk in young adults using the 2017 American college of cardiology/American heart association blood pressure guideline: a longitudinal cohort study. J Hypertens. (2023) 41:271–9. 10.1097/HJH.0000000000003325 36583352

[8] Li J Zhao F Wang Y Chen J Tao J Tian G Gut microbiota dysbiosis contributes to the development of hypertension. Microbiome. (2017) 5:14. 10.1186/s40168-016-0222-x 28143587 PMC5286796

[9] Li J Yang X Zhou X Cai J. The role and mechanism of intestinal Flora in blood pressure regulation and hypertension development. Antioxid Redox Signal. (2021) 34:811–30. 10.1089/ars.2020.8104 32316741

[10] Kurilshikov A van den Munckhof ICL Chen L Bonder MJ Schraa K Rutten JHW Gut microbial associations to plasma metabolites linked to cardiovascular phenotypes and risk. Circ Res. (2019) 124:1808–20. 10.1161/CIRCRESAHA.118.314642 30971183

[11] Nakai M Ribeiro RV Stevens BR Gill P Muralitharan RR Yiallourou S Essential hypertension is associated with changes in gut microbial metabolic pathways: a multisite analysis of ambulatory blood pressure. Hypertension. (2021) 78:804–15. 10.1161/HYPERTENSIONAHA.121.17288 34333988

[12] Avery EG Bartolomaeus H Maifeld A Marko L Wiig H Wilck N The gut microbiome in hypertension: recent advances and future perspectives. Circ Res. (2021) 128:934–50. 10.1161/CIRCRESAHA.121.318065 33793332

[13] Chu H Duan Y Yang L Schnabl B. Small metabolites, possible big changes: a microbiota-centered view of non-alcoholic fatty liver disease. Gut. (2019) 68:359–70. 10.1136/gutjnl-2018-316307 30171065

[14] Visconti A Le Roy CI Rosa F Rossi N Martin TC Mohney RP Interplay between the human gut microbiome and host metabolism. Nat Commun. (2019) 10:4505. 10.1038/s41467-019-12476-z 31582752 PMC6776654

[15] Jiang S Shui Y Cui Y Tang C Wang X Qiu X Gut microbiota dependent trimethylamine N-oxide aggravates angiotensin II-induced hypertension. Redox Biol. (2021) 46:102115. 10.1016/j.redox.2021.102115 34474396 PMC8408632

[16] Walejko JM Kim S Goel R Handberg EM Richards EM Pepine CJ Gut microbiota and serum metabolite differences in African Americans and white Americans with high blood pressure. Int J Cardiol. (2018) 271:336–9. 10.1016/j.ijcard.2018.04.074 30049487 PMC6143419

[17] Chachaj A Matkowski R Grobner G Szuba A Dudka I. Metabolomics of interstitial fluid, plasma and urine in patients with arterial hypertension: new insights into the underlying mechanisms. Diagnostics. (2020) 10:936. 10.3390/diagnostics10110936 33187152 PMC7698256

[18] Dan X Mushi Z Baili W Han L Enqi W Huanhu Z Differential analysis of hypertension-associated intestinal microbiota. Int J Med Sci. (2019) 16:872–81. 10.7150/ijms.29322 31337961 PMC6643114

[19] Wang P Dong Y Zuo K Han C Jiao J Yang X Characteristics and variation of fecal bacterial communities and functions in isolated systolic and diastolic hypertensive patients. BMC Microbiol. (2021) 21:128. 10.1186/s12866-021-02195-1 33902467 PMC8077764

[20] Li FR He Y Yang HL Liu HM Zhou R Chen GC Isolated systolic and diastolic hypertension by the 2017 American college of cardiology/American heart association guidelines and risk of cardiovascular disease: a large prospective cohort study. J Hypertens. (2021) 39:1594–601. 10.1097/HJH.0000000000002805 33560057

[21] Dunn WB Broadhurst D Begley P Zelena E Francis-McIntyre S Anderson N Procedures for large-scale metabolic profiling of serum and plasma using gas chromatography and liquid chromatography coupled to mass spectrometry. Nat Protoc. (2011) 6:1060–83. 10.1038/nprot.2011.335 21720319

[22] Kanehisa M Sato Y Kawashima M Furumichi M Tanabe M. KEGG as a reference resource for gene and protein annotation. Nucleic Acids Res. (2016) 44:D457–62. 10.1093/nar/gkv1070 26476454 PMC4702792

[23] Sreekumar A Poisson LM Rajendiran TM Khan AP Cao Q Yu J Metabolomic profiles delineate potential role for sarcosine in prostate cancer progression. Nature. (2009) 457:910–4. 10.1038/nature07762 19212411 PMC2724746

[24] Aggio RB Ruggiero K Villas-Boas SG. Pathway Activity Profiling (PAPi): from the metabolite profile to the metabolic pathway activity. Bioinformatics. (2010) 26:2969–76. 10.1093/bioinformatics/btq567 20929912

[25] Trygg J Wold S. Orthogonal projections to latent structures (O-PLS). J Chemom. (2002) 16:119–28. 10.1002/cem.695

[26] Li J Gao Q Ma Y Deng Y Li S Shi N Causality of opportunistic pathogen *Klebsiella pneumoniae* to hypertension development. Hypertension. (2022) 79:2743–54. 10.1161/HYPERTENSIONAHA.122.18878 36259407

[27] Stępniewska J Dołęgowska B Puchałowicz K Gołembiewska E Ciechanowski K. Bioactive lipids derived from arachidonic acid metabolism in different types of renal replacement therapy. Chem Phys Lipids. (2017) 206:71–7. 10.1016/j.chemphyslip.2017.05.003 28533146

[28] Zhang M Jia S Liu Y Liu Y Li S Bo L Metabonomics analysis of kidneys in rats administered with chronic low-dose cadmium by ultra-performance liquid chromatography-mass spectrometry. J Appl Toxicol. (2019) 39:441–50. 10.1002/jat.3735 30325046

[29] Li Q Rempel JD Ball TB Aukema H Minuk GY. Plasma oxylipins levels in nonalcoholic fatty liver disease. Dig Dis Sci. (2020) 65:3605–13. 10.1007/s10620-020-06095-8 31997053

[30] Caussy C Chuang JC Billin A Hu T Wang Y Subramanian GM Plasma eicosanoids as noninvasive biomarkers of liver fibrosis in patients with nonalcoholic steatohepatitis. Therap Adv Gastroenterol. (2020) 13:1756284820923904. 10.1177/1756284820923904 32523627 PMC7257854

[31] de la Cuesta-Zuluaga J Mueller NT Álvarez-Quintero R Velásquez-Mejía EP Sierra JA Corrales-Agudelo V Higher fecal short-chain fatty acid levels are associated with gut microbiome dysbiosis, obesity, hypertension and cardiometabolic disease risk factors. Nutrients. (2018) 11:51. 10.3390/nu11010051 30591685 PMC6356834

[32] Shoaie S Ghaffari P Kovatcheva-Datchary P Mardinoglu A Sen P Pujos-Guillot E Quantifying diet-induced metabolic changes of the human gut microbiome. Cell Metab. (2015) 22:320–31. 10.1016/j.cmet.2015.07.001 26244934

[33] Reuter SE Evans AM. Carnitine and acylcarnitines: pharmacokinetic, pharmacological and clinical aspects. Clin Pharmacokinet. (2012) 51:553–72. 10.1007/bf03261931 22804748

[34] Kang M Yoo HJ Kim M Kim M Lee JH. Metabolomics identifies increases in the acylcarnitine profiles in the plasma of overweight subjects in response to mild weight loss: a randomized, controlled design study. Lipids Health Dis. (2018) 17:237. 10.1186/s12944-018-0887-1 30322392 PMC6190541

[35] Rinaldo P Matern D Bennett MJ. Fatty acid oxidation disorders. Annu Rev Physiol. (2002) 64:477–502. 10.1146/annurev.physiol.64.082201.154705 11826276

[36] Baek SH Kim M Kim M Kang M Yoo HJ Lee NH Metabolites distinguishing visceral fat obesity and atherogenic traits in individuals with overweight. Obesity. (2017) 25:323–31. 10.1002/oby.21724 28000430

[37] Luo N Craig D Ilkayeva O Muehlbauer M Kraus WE Newgard CB Plasma acylcarnitines are associated with pulmonary hypertension. Pulm Circ. (2017) 7:211–8. 10.1086/690554 28680580 PMC5448546

[38] Hoque R Farooq A Ghani A Gorelick F Mehal WZ. Lactate reduces liver and pancreatic injury in toll-like receptor- and inflammasome-mediated inflammation via GPR81-mediated suppression of innate immunity. Gastroenterology. (2014) 146:1763–74. 10.1053/j.gastro.2014.03.014 24657625 PMC4104305

[39] Sun S Li H Chen J Qian Q. Lactic acid: no longer an inert and end-product of glycolysis. Physiology. (2017) 32:453–63. 10.1152/physiol.00016.2017 29021365

[40] Shen Z Jiang L Yuan Y Deng T Zheng YR Zhao YY Inhibition of G protein-coupled receptor 81 (GPR81) protects against ischemic brain injury. CNS Neurosci Ther. (2015) 21:271–9. 10.1111/cns.12362 25495836 PMC6495224

[41] Walenta S Mueller-Klieser WF. Lactate: mirror and motor of tumor malignancy. Semin Radiat Oncol. (2004) 14:267–74. 10.1016/j.semradonc.2004.04.004 15254870

[42] Aichler M Borgmann D Krumsiek J Buck A MacDonald PE Fox JEM N-acyl taurines and acylcarnitines cause an imbalance in insulin synthesis and secretion provoking β cell dysfunction in type 2 diabetes. Cell Metab. (2017) 25:1334–47.e4. 10.1016/j.cmet.2017.04.012 28591636

[43] Dave AM Genaro-Mattos TC Korade Z Peeples ES. Neonatal hypoxic-ischemic brain injury alters brain acylcarnitine levels in a mouse model. Metabolites. (2022) 12:467. 10.3390/metabo12050467 PMC9143624 35629971

[44] Chouvarine P Giera M Kastenmüller G Artati A Adamski J Bertram H Trans-right ventricle and transpulmonary metabolite gradients in human pulmonary arterial hypertension. Heart. (2020) 106:1332–41. 10.1136/heartjnl-2019-315900 32079620 PMC7476282

