# Supplementary material for: A cross-sectional study identifying disparities in serum metabolic profiles among hypertensive patients with ISH, IDH and SDH subtypes
Source: Front Cardiovasc Med. 2023 May 4;10:1102754. doi: 10.3389/fcvm.2023.1102754 (PMC10192909; doi:10.3389/fcvm.2023.1102754)
Supplement: Supplementary file 1 [file Table1.doc]

Table S1. General characteristics by normal BP and HTN subtype according to the ACC/AHA guidelines.

| Characteristics | Nor | ISH | IDH | SDH | P1-Value  (Nor vs. ISH) | P2-Value  (Nor vs. IDH) | P3-Value  (Nor vs. SDH) | P4-Value  (ISH vs. IDH) | P5-Value  (ISH vs. SDH) | P6-Value  (IDH vs. SDH) |
| --- | --- | --- | --- | --- | --- | --- | --- | --- | --- | --- |
| Number | 13 | 11 | 27 | 68 |  |  |  |  |  |  |
| Age, years | 56.0 (48.5-60.0) | 59.0 (52.0-63.0) | 51.0 (49.0-54.0) | 53.0 (49.0-57.0) | 0.331 | 0.289 | 0.545 | 0.016 | 0.030 | 0.202 |
| Male/female sex | 6/7 | 11/0 | 24/3 | 64/4 | 0.005 | 0.006 | 0.000 | 0.542 | 1.000 | 0.401 |
| Systolic BP, mmHg | 115.3 (106.0-118.2) | 139.0 (130.0-156.7) | 120.0 (117.0-120.0) | 145.5 (139.2-159.8) | 0.000 | 0.001 | 0.000 | 0.000 | 0.187 | 0.000 |
| Diastolic BP, mmHg | 70.7 (68.0-75.3) | 77.0 (72.7-79.0) | 81.3 (80.0-85.0) | 93.5 (89.1-100.0) | 0.042 | 0.000 | 0.000 | 0.000 | 0.000 | 0.000 |
| HR, bmp | 68.5 (65.5-71.0) | 69.5 (60.5-76.5) | 72.0 (68.0-75.5) | 72.0 (67.5-81.5) | 0.633 | 0.148 | 0.065 | 0.456 | 0.285 | 0.557 |
| BMI, kg/m^2^ | 24.0 (21.4-28.6) | 24.7 (22.5-26.5) | 25.8 (24.2-27.2) | 26.0 (23.9-27.8) | 0.832 | 0.286 | 0.218 | 0.397 | 0.254 | 0.561 |
| Uric acid, mmol/L | 256.0 (188.8-349.5) | 359.0 (232.0-453.0) | 360.5 (306.8-393.5) | 358.5 (309.3-413.0) | 0.190 | 0.023 | 0.009 | 0.895 | 0.600 | 0.670 |
| Creatinine, mmol/L | 64.0 (58.5-72.5) | 72.0 (62.0-102.6) | 71.0 (64.0-76.0) | 70.0 (59.3-80.8) | 0.134 | 0.263 | 0.358 | 0.637 | 0.406 | 0.885 |
| FBG, mmol/L | 5.2 (4.7-5.5) | 5.8 (5.5-6.1) | 5.2 (4.9-5.7) | 5.7 (5.1-6.2) | 0.000 | 0.362 | 0.009 | 0.011 | 0.402 | 0.067 |
| TC, mmol/L | 5.3 (4.7-5.9) | 5.6 (4.9-6.1) | 5.1 (4.7-3.0) | 5.2 (4.7-5.9) | 0.651 | 0.668 | 0.776 | 0.424 | 0.393 | 0.840 |
| Triglyceride, mmol/L | 1.0 (0.9-1.7) | 1.4 (1.0-1.92) | 1.3 (1.0-2.4) | 1.6 (1.0-2.5) | 0.347 | 0.161 | 0.114 | 0.856 | 0.905 | 0.707 |
| HDLC, mmol/L | 1.2 (1.1-1.5) | 1.4 (1.2-1.9) | 1.2 (1.1-1.4) | 1.3 (1.1-1.4) | 0.347 | 0.878 | 0.743 | 0.176 | 0.203 | 0.590 |
| LDLC, mmol/L | 2.6 (2.2-3.6) | 2.9 (2.0-3.2) | 2.6 (2.0-2.8) | 2.4 (2.1-3.0) | 0.847 | 0.612 | 0.863 | 0.214 | 0.496 | 0.444 |
| Hemoglobin, g/L | 147.0 (129.5-156.3) | 160.0 (157.0-163.0) | 159.0 (149.0-163.0) | 157.0 (151.3-165.8) | 0.039 | 0.057 | 0.010 | 0.612 | 0.880 | 0.661 |
| Blood platelet, *10^9/L | 255.0 (186.5-298.3) | 235.0 (184.0-263.0) | 252.0 (189.0-277.0) | 220.5 (185.3-264.0) | 0.379 | 0.482 | 0.311 | 0.424 | 0.899 | 0.618 |
| white blood cell, *10^9/L | 6.1 (5.3-6.7) | 6.3 (5.9-7.1) | 6.3 (5.1-8.8) | 6.3 (5.3-7.2) | 0.379 | 0.482 | 0.645 | 0.942 | 0.515 | 0.393 |

P1-value: Nor versus ISH; P2-value: Nor versus IDH; P3-value: Nor versus SDH; P4-value: ISH versus IDH; P5-Value: ISH versus SDH; P6-Value: IDH versus SDH. Nor: Healthy controls; ISH: Isolated systolic HTN; IDH: Isolated diastolic HTN; SDH: Systolic diastolic HTN; HTN: Hypertension; BP: Blood pressure; HR: Heart rate; BMI: body mass index; FBG: fasting blood glucose; TC: total cholesterol; LDLC: Low-density lipoprotein cholesterol; HDLC: High-density lipoprotein cholesterol.
