# Supplementary material for: A cross-sectional study identifying disparities in serum metabolic profiles among hypertensive patients with ISH, IDH and SDH subtypes
Source: Front Cardiovasc Med. 2023 May 4;10:1102754. doi: 10.3389/fcvm.2023.1102754 (PMC10192909; doi:10.3389/fcvm.2023.1102754)
Supplement: Supplementary file 2 [file Table2.doc]

Supplementary Tables

| Table S2. Multivariable Linear Regression shows the independent strength of association between BP status and serum metabolic profiles (Nor VS. ISH) under negative mode. | | | | | | | | | | |
| --- | --- | --- | --- | --- | --- | --- | --- | --- | --- | --- |
|  | 15(S)-Hydroxyeicosatrienoic acid | | 12-Hydroxydodecanoic acid | | Hexadecanedioic acid | | 7-Ketocholesterol | | 3-Hydroxyvalproic acid | |
|  | beta | P | beta | P | beta | P | beta | P | beta | P |
| ISH | 0.00001030 | 0.083 | 0.00007995 | 0.052 | 0.00007725 | 0.037 | 0.00025680 | 0.002 | 0.00001430 | 0.6452 |
| female | -6.24766E-8 | 0.990 | 0.00002367 | 0.480 | 0.00001887 | 0.528 | -0.00002410 | 0.700 | -0.00003045 | 0.2628 |
| FBG | -7.63903E-7 | 0.792 | 0.00000302 | 0.879 | -0.00000606 | 0.732 | -0.00005195 | 0.173 | 0.00000371 | 0.8146 |
| Hemoglobin | 3.54175E-8 | 0.772 | 0.00000117 | 0.174 | 7.017706E-7 | 0.353 | 1.435309E-7 | 0.927 | -5.30453E-7 | 0.4315 |

Nor: Healthy controls; ISH: Isolated systolic hypertension; BP: Blood pressure; FBG: fasting blood glucose.

| Table S3. Multivariable Linear Regression shows the independent strength of association between BP status and serum metabolic profiles (Nor VS. ISH) under positive mode. | | | | | | | | | | |
| --- | --- | --- | --- | --- | --- | --- | --- | --- | --- | --- |
|  | L-Methionine sulfoxide | | LysoPE(18:0/0:0) | | 13-HODE | | LysoPC(P-16:0/0:0) | | Palmitelaidic acid | |
|  | beta | P | beta | P | beta | P | beta | P | beta | P |
| ISH | -0.00010819 | 0.201 | -0.00012291 | 0.274 | 0.00001366 | 0.029 | -0.00012932 | 0.347 | 0.00001741 | 0.013 |
|  |  |  |  |  |  |  |  |  |  |  |
| female | -0.00010496 | 0.153 | 0.00001051 | 0.912 | -3.56E-08 | 0.994 | -0.00029222 | 0.022 | 0.00000862 | 0.130 |
| FBG | -0.00001947 | 0.645 | -0.00006288 | 0.273 | -0.00000196 | 0.509 | -0.00004052 | 0.561 | 1.22E-07 | 0.970 |
| Hemoglobin | -0.00000191 | 0.292 | 0.00000227 | 0.346 | -2.26E-07 | 0.083 | -0.00000746 | 0.020 | 1.67E-07 | 0.234 |

Nor: Healthy controls; ISH: Isolated systolic hypertension; BP: Blood pressure; FBG: fasting blood glucose.

| Table S4. Multivariable Linear Regression shows the independent strength of association between BP status and serum metabolic profiles (Nor VS. IDH) under negative mode. | | | | | | | | | | |
| --- | --- | --- | --- | --- | --- | --- | --- | --- | --- | --- |
|  | Ketoleucine | | L-Glutamine | | Indolelactic acid | | Levulinic acid | | Hexadecanedioic acid | |
|  | beta | P | beta | P | beta | P | beta | P | beta | P |
| IDH | 0.00073485 | 0.047 | -0.00006365 | 0.104 | -0.00003885 | 0.688 | 0.00018828 | 0.034 | 0.00004910 | 0.004 |
| Female | -0.00043382 | 0.142 | 0.00007062 | 0.030 | -0.00011091 | 0.166 | -0.00003675 | 0.597 | -0.00001387 | 0.280 |
| Uric acid | -0.00000157 | 0.214 | -2.68636E-9 | 0.9840 | -1.74823E-7 | 0.606 | -5.22916E-7 | 0.087 | -5.18826E-8 | 0.346 |

Nor: Healthy controls; IDH: Isolated diastolic hypertension; BP: Blood pressure.

| Table S5. Multivariable Linear Regression shows the independent strength of association between BP status and serum metabolic profiles (Nor VS. IDH) under positive mode. | | | | | | | | | | |
| --- | --- | --- | --- | --- | --- | --- | --- | --- | --- | --- |
|  | L-Glutamic acid | | N-Arachidonoyl Dopamine | | Indolelactic acid | | L-Serine | | N-lauroylglycine | |
|  | beta | P | beta | P | beta | P | beta | P | beta | P |
| IDH | 0.00003803 | 0.008 | -0.00000677 | 0.164 | 0.00003377 | 0.468 | -0.00001045 | 0.888 | -4.90E-07 | 0.431 |
| Female | -0.00000227 | 0.834 | -1.54E-07 | 0.969 | -0.00003507 | 0.355 | 0.000089 | 0.148 | -6.25E-08 | 0.901 |
| Uric acid | 7.28E-08 | 0.126 | -5.40E-09 | 0.747 | -8.11E-08 | 0.617 | 4.07E-08 | 0.875 | -1.82E-09 | 0.402 |

Nor: Healthy controls; IDH: Isolated diastolic hypertension; BP: Blood pressure.

| Table S6. Multivariable Linear Regression shows the independent strength of association between BP status and serum metabolic profiles (Nor VS. SDH) under negative mode. | | | | | | | | | | |
| --- | --- | --- | --- | --- | --- | --- | --- | --- | --- | --- |
|  | Urobilinogen | | Indolelactic acid | | 7-Ketocholesterol | | 12-Hydroxydodecanoic acid | | Hexadecanedioic acid | |
|  | beta | P | beta | P | beta | P | beta | P | beta | P |
| SDH | 0.00001640 | 0.209 | 0.00006204 | 0.377 | 0.00001363 | 0.738 | 0.00000868 | 0.687 | 0.00000474 | 0.765 |
| Female | -0.00000979 | 0.429 | -0.00009109 | 0.175 | -0.00005410 | 0.166 | -0.00003480 | 0.093 | -0.00003267 | 0.033 |
| Uric acid | 2.801886E-8 | 0.397 | 1.411427E-7 | 0.429 | -5.46689E-9 | 0.958 | -2.11675E-8 | 0.699 | -2.5273E-8 | 0.530 |
| FBG | -0.00000154 | 0.509 | -0.00000753 | 0.550 | 0.00000711 | 0.333 | 0.00000757 | 0.054 | 0.00000317 | 0.268 |
| Hemoglobin | -2.61746E-8 | 0.927 | -0.00000148 | 0.339 | -3.7063E-7 | 0.681 | -8.96182E-7 | 0.063 | -7.66386E-7 | 0.031 |

Nor: Healthy controls; SDH: Systolic diastolic hypertension; BP: Blood pressure; FBG: fasting blood glucose.

| Table S7. Multivariable Linear Regression shows the independent strength of association between BP status and serum metabolic profiles (Nor VS. SDH) under positive mode. | | | | | | | | | | |
| --- | --- | --- | --- | --- | --- | --- | --- | --- | --- | --- |
|  | Palmitelaidic acid | | Calcitriol | | L-Methionine sulfoxide | | N-Arachidonoyl Dopamine | | Linoleyl Carnitine | |
|  | beta | P | beta | P | beta | P | beta | P | beta | P |
| SDH | 3.88E-07 | 0.951 | 0.00000716 | 0.716 | -0.00005409 | 0.205 | -0.00000583 | 0.146 | 0.00010731 | 0.658 |
| Female | -0.00000166 | 0.781 | -9.72E-07 | 0.959 | 0.00000413 | 0.918 | 1.91E-07 | 0.960 | 0.00018121 | 0.433 |
| Uric acid | -2.26E-08 | 0.158 | 3.12E-08 | 0.532 | 9.07E-08 | 0.401 | 1.34E-08 | 0.186 | 0.00000148 | 0.019 |
| FBG | 0.00000141 | 0.211 | 0.00000738 | 0.040 | 0.000007 | 0.360 | -5.13E-07 | 0.473 | 0.00002827 | 0.517 |
| Hemoglobin | 3.69E-08 | 0.790 | 8.02E-08 | 0.854 | 2.99E-07 | 0.750 | -1.09E-07 | 0.215 | 0.00000908 | 0.094 |

Nor: Healthy controls; SDH: Systolic diastolic hypertension; BP: Blood pressure; FBG: fasting blood glucose.

| Table S8. Multivariable Linear Regression shows the independent strength of association between BP status and serum metabolic profiles (ISH VS. IDH) under negative mode. | | | | | | | | | | |
| --- | --- | --- | --- | --- | --- | --- | --- | --- | --- | --- |
|  | LysoPC(14:0/0:0) | | L-Homoserine | | Maleic acid | | 12-Hydroxydodecanoic acid | | Estrone glucuronide | |
|  | beta | P | beta | P | beta | P | beta | P | beta | P |
| IDH | -0.00009960 | 0.127 | 0.00002951 | 0.005 | -0.00000202 | 0.392 | 0.00004933 | 0.112 | 0.00002637 | 0.002 |
| Age | -9.81974E-7 | 0.690 | -4.50322E-7 | 0.238 | -1.59421E-07 | 0.086 | -1.25E-07 | 0.915 | -5.95E-07 | 0.054 |
| FBG | -0.00000931 | 0.586 | 0.00000475 | 0.077 | 8.80296E-08 | 0.888 | 0.00000444 | 0.583 | 0.00000281 | 0.183 |

ISH: Isolated systolic hypertension; IDH: Isolated diastolic hypertension; BP: Blood pressure; FBG: fasting blood glucose.

| Table S9. Multivariable Linear Regression shows the independent strength of association between BP status and serum metabolic profiles (ISH VS. IDH) under positive mode. | | | | | | | | | | |
| --- | --- | --- | --- | --- | --- | --- | --- | --- | --- | --- |
|  | Sphingosine-1-phosphate (d16:1) | | LysoPE(18:0/0:0) | | Octadecanamide | | L-Acetylcarnitine | | L-Methionine | |
|  | beta | P | beta | P | beta | P | beta | P | beta | P |
| IDH | -0.00002573 | 0.135 | -0.00023816 | 0.085 | 0.00001793 | 0.026 | 0.00006472 | 0.810 | 0.00000135 | 0.265 |
| Age | 4.36E-08 | 0.946 | -5.25E-07 | 0.919 | -4.13E-07 | 0.171 | 0.0000142 | 0.177 | 5.04E-08 | 0.280 |
| FBG | 0.00000517 | 0.256 | 0.00000645 | 0.857 | 0.00000137 | 0.506 | 0.00018468 | 0.015 | -2.79E-07 | 0.386 |

ISH: Isolated systolic hypertension; IDH: Isolated diastolic hypertension; BP: Blood pressure; FBG: fasting blood glucose.

| Table S10. Multivariable Linear Regression shows the independent strength of association between BP status and serum metabolic profiles (ISH VS. SDH) under negative mode | | | | | | | | | | |
| --- | --- | --- | --- | --- | --- | --- | --- | --- | --- | --- |
|  | Glyoxylic acid | | L-Homoserine | | Malic acid | | Deoxycholic acid glycine conjugate | | L-Lactic acid | |
|  | beta | P | beta | P | beta | P | beta | P | beta | P |
| SDH | 0.00003048 | 0.302 | -0.00001183 | 0.028 | 0.00002999 | 0.325 | 0.00000762 | 0.045 | 0.00132 | 0.110 |
| Age | -7.67E-07 | 0.535 | -4.47E-08 | 0.840 | -7.36E-07 | 0.564 | -2.00E-07 | 0.207 | -0.00001083 | 0.754 |

ISH: Isolated systolic hypertension; SDH: Systolic diastolic hypertension; BP: Blood pressure.

| Table S11. Multivariable Linear Regression shows the independent strength of association between BP status and serum metabolic profiles (ISH VS. SDH) under positive mode. | | | | | | | | | | |
| --- | --- | --- | --- | --- | --- | --- | --- | --- | --- | --- |
|  | Ornithine | | 2-Methylbutyroylcarnitine | | 7-Ketodeoxycholic acid | | 3,4-Dihydroxymandelic acid | | Caprylic acid | |
|  | beta | P | beta | P | beta | P | beta | P | beta | P |
| SDH | 0.00000539 | <0.001 | -4.919E-05 | 0.101 | 0.00000229 | 0.051 | -1.677E-05 | 0.926 | 2.58E-07 | 0.397 |
| Age | 1.07E-07 | 0.064 | -4.74E-07 | 0.704 | 6.73E-08 | 0.168 | 0.0000045 | 0.554 | 7.38E-09 | 0.562 |

ISH: Isolated systolic hypertension; SDH: Systolic diastolic hypertension; BP: Blood press
