# Supplementary material for: A cross-sectional study identifying disparities in serum metabolic profiles among hypertensive patients with ISH, IDH and SDH subtypes
Source: Front Cardiovasc Med. 2023 May 4;10:1102754. doi: 10.3389/fcvm.2023.1102754 (PMC10192909; doi:10.3389/fcvm.2023.1102754)
Supplement: Supplementary file 3 [file Datasheet1.doc]

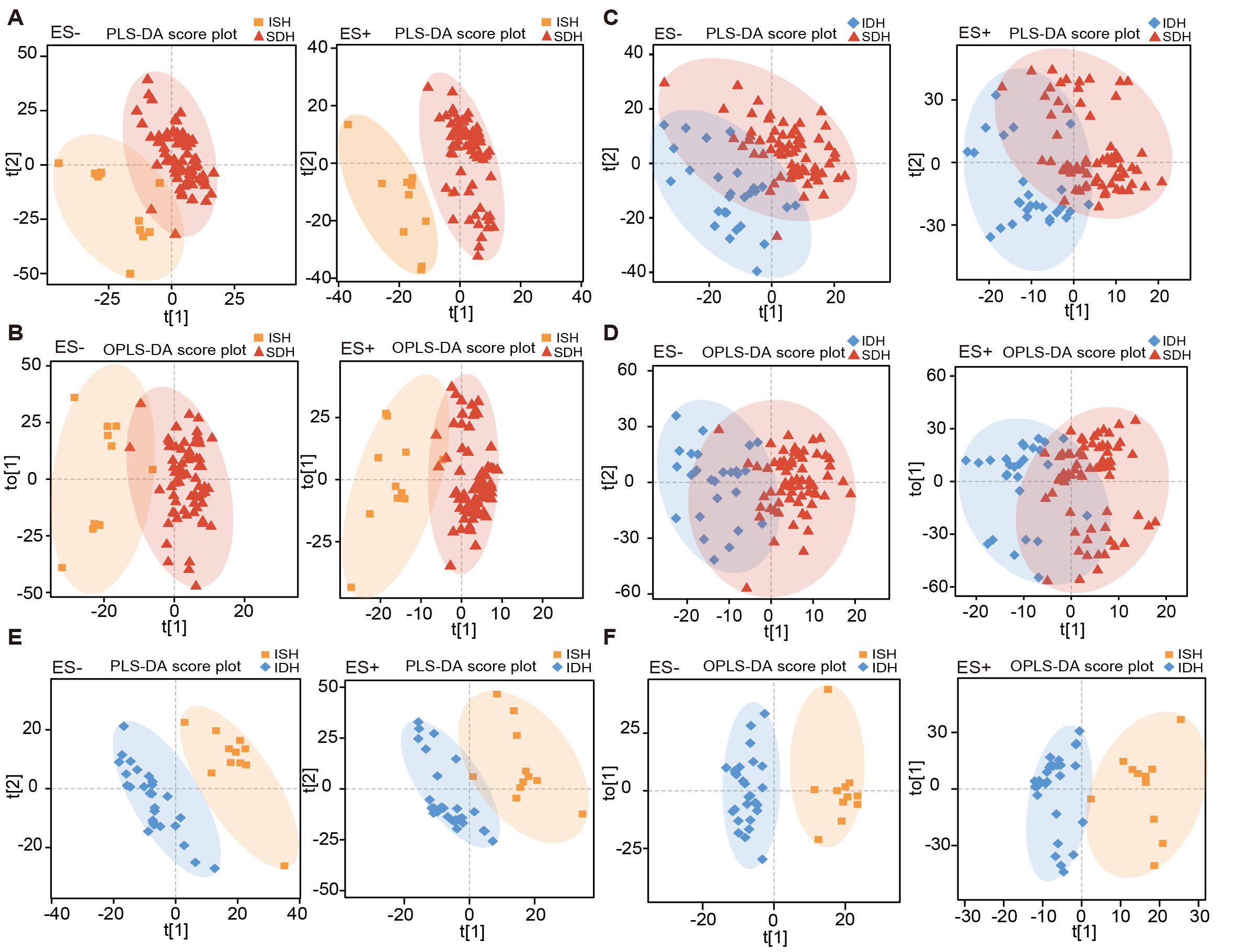
**Supplementary Figure Legends**

**Figure S1. Patients with ISH, IDH or SDH showed distinct serum metabolic characteristics when compared with each other.** **A,** Comparison of the metabolic signatures in ISH and SDH according to PLS-DA score plots in both negative (ES-) and positive (ES+) mode. **B,** OPLS-DA score plots describing the disparate metabolic patterns between ISH patients and SDHs. The supervised multiple regression analysis for identifying discernible profiles in ES- was indicated in left, and ES+ was in right. **C,** PLS-DA score plots of both ES- and ES+ mode distinguished serum metabolomic data of IDH patients from SDH. **D,** Supervise multiple regression analysis of OPLS-DA was performed to identify the heterogeneity between IDH and SDH in ES- and ES+ in scatter plots. **E,** Serum metabolomic data of patients suffered from IDH were separated from ISH with PLS-DA score plots in ES- and ES+ mode. **F,** Supervise multiple regression analysis of OPLS-DA was performed to identify the heterogeneity between IDH and ISH in ES- and ES+ in scatter plots.


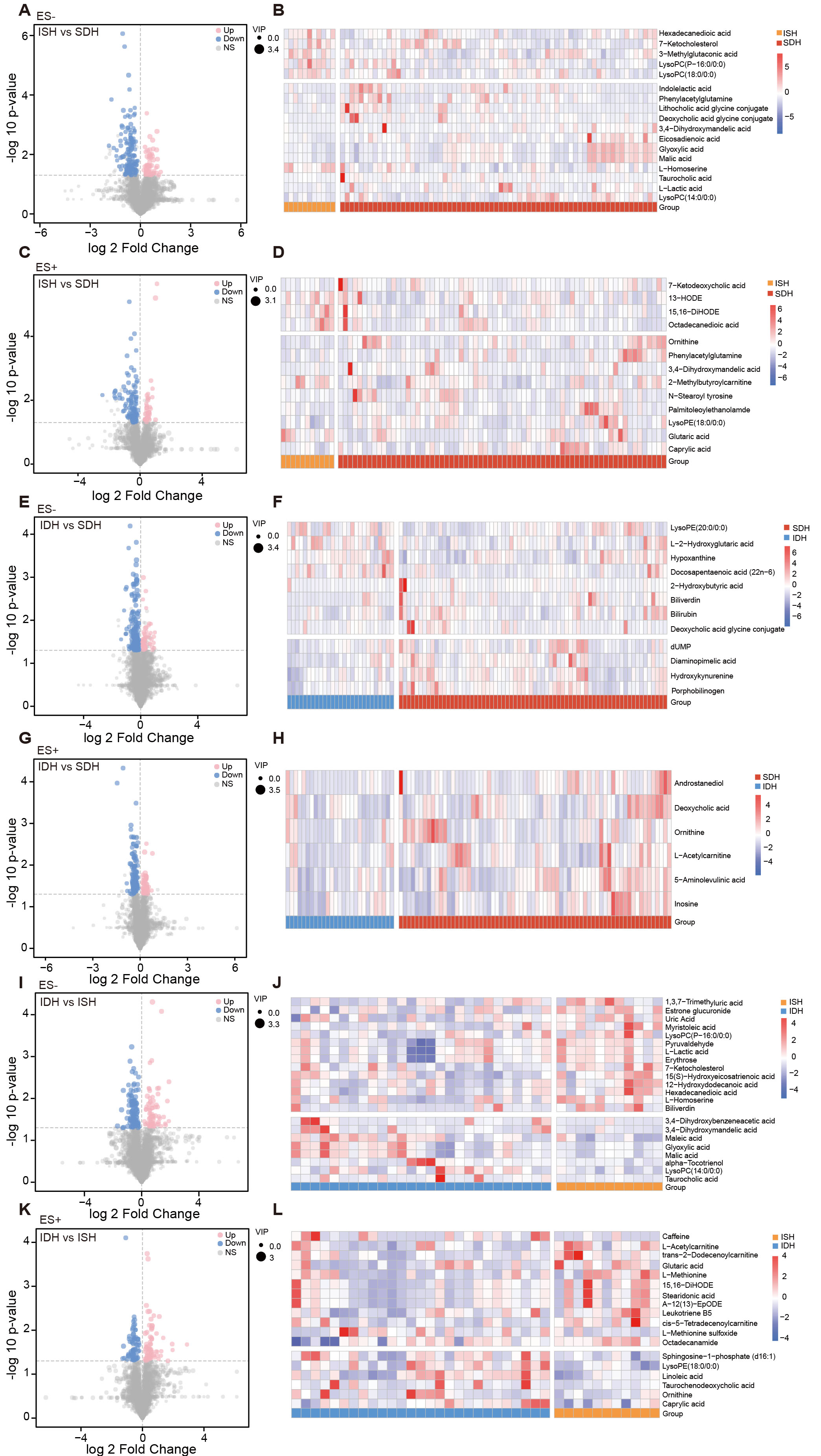
**Figure S2.** **The significantly differential metabolites among ISH, IDH and SDH patients. A, C,** Volcano plots illustrating the fold changes, p values and VIP of each detected metabolic peaks in ISH and SDH group under negative and positive mode. For significantly varied peaks in ISH, the threshold of VIP in OPLS-DA model was >1, and the relative abundance was additionally screened with P values<0.05 from two-tailed Student’s t-test. Dots represented metabolites, blue denoted down-regulated, pink represented those up-regulated in ISH, and grey were not significant different peaks and dot size showed the value of VIP. **B, D,** Relative abundance of each metabolite identified to be prominently altered in ISH was described in heatmaps. Only those successfully identified were shown. B shows those detected in ES-, and D displays those in ES+. **E, G,** Metabolites shifted between IDH and SDH patients were detected with volcano plots. E was derived based on metabolomics data in ES- mode, and G was in ES+ mode. The metabolic peaks with VIP >1 in OPLS-DA model, and P value <0.05 tested with two-tailed Student’s t-test were considered to be significantly distinct in IDH. Dots denoted metabolites detected, blue ones were those reduced in IDH, and pink represented those enriched in IDH. The values of VIP were displayed with the dot size, and fold change were shown. **F, H,** Among the markedly varied metabolites between IDH and SDH group, the relative abundance of those successfully identified were further shown in hierarchical cluster analysis heat-maps. F for those in ES-, and H for those in ES+. **I, K,** Volcano plots identifying the discriminative metabolites between IDH and ISH was conducted with the OPLS-DA model using a threshold of VIP >1, and the two-tailed Student’s t-test with P values <0.05. I was based on ES- mode, and K was in ES+ mode. The dots represented various metabolites, and color denoted significantly down-regulated or up-regulated in IDH. Dot size was depicted according to the value of VIP. **J, L,** Of the varied metabolites, heatmaps illustrated comparison for the relative abundance of those identified metabolites with significant disparate levels between IDH patients and ISH.


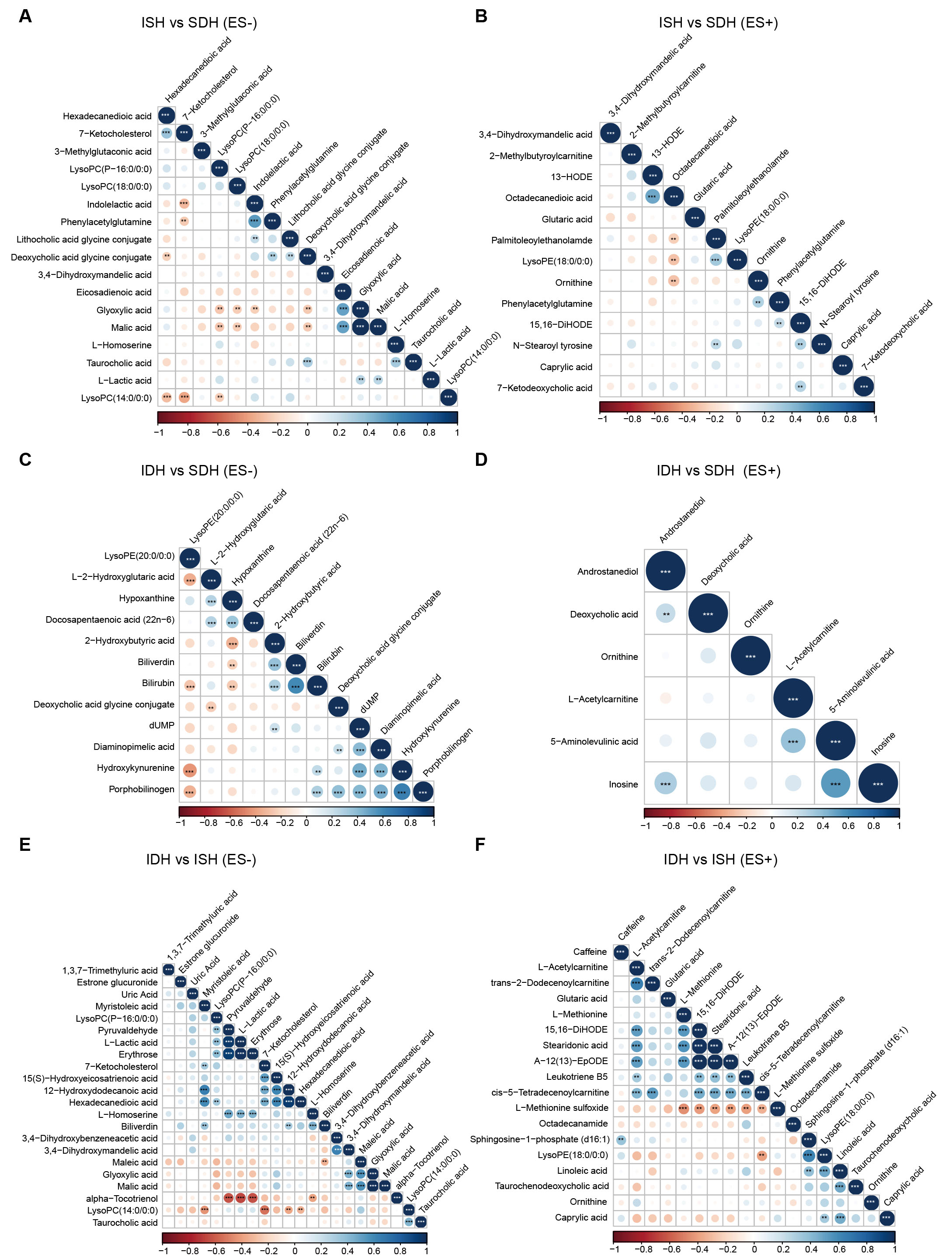
**Figure S3.** **Correlation of the metabolites distinct between groups was identified via Spearman analysis. A-B,** Co-abundance correlation of the distinct metabolites identified in ISH patients as compared with SDHs under ES- and ES+ mode, respectively. **C-D,** Variants with significant different metabolic compositions between IDH and SDH in negative and positive mode were shown to be related with each other. **E-F,** Heat map depicting the potential correlation of differently abundant metabolites identified between IDH and ISH. E for those in ES-, and F for ES+. Negative correlation was depicted in orange and positive correlation was in blue. The shade of and size of dots represented the correlation coefficient. *, p < 0.05; **, p < 0.01; ***, p < 0.001; derived from Spearman’s correlation.


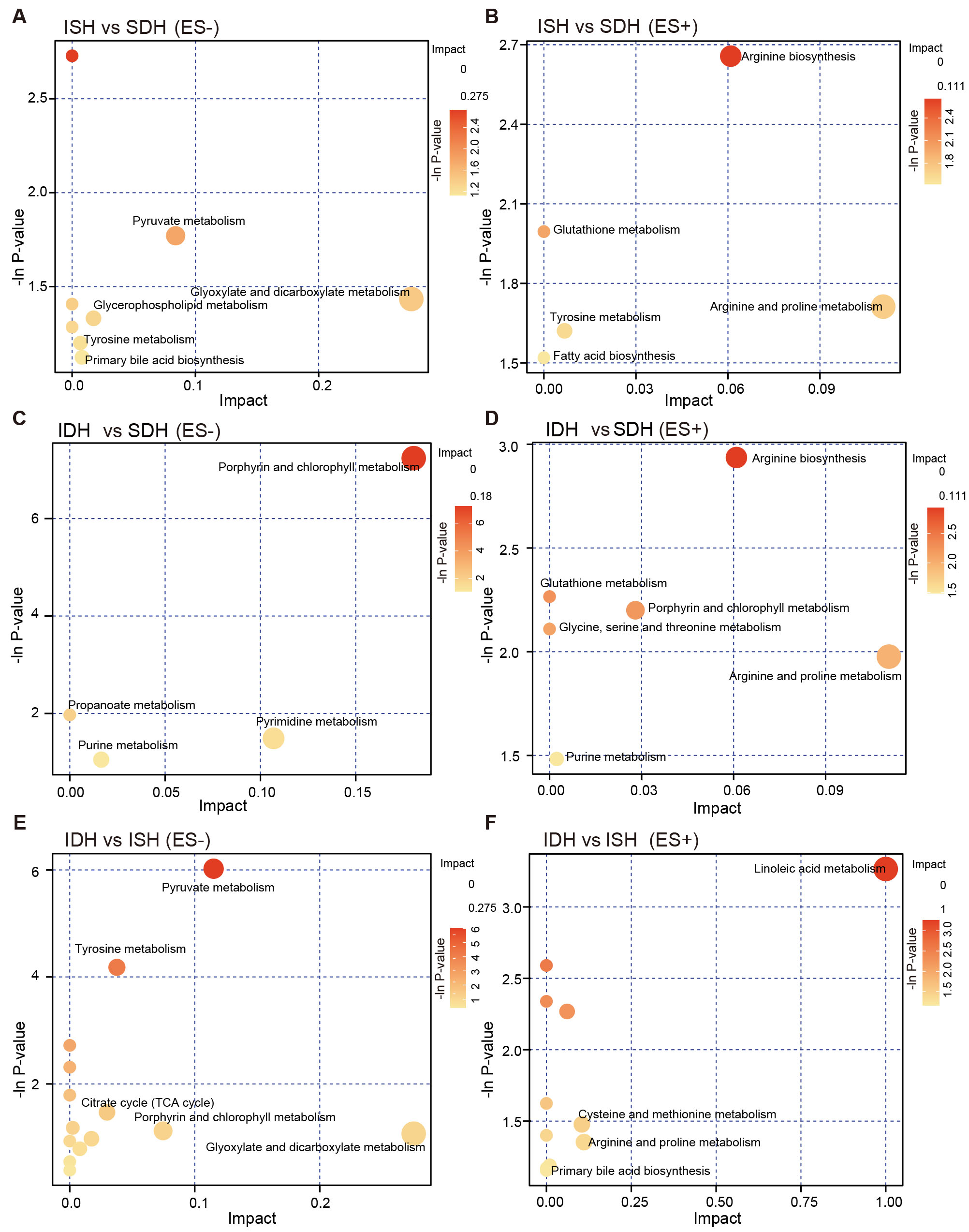
**Figure S4. The differentially enriched metabolites among ISH, IDH, SDH patients functioned in distinct KEGG pathways. A-B,** Bubble plots in ES- and ES+ mode showed the enriched metabolic pathways of altered metabolic compounds between ISH and SDH group. **C-D,** Metabolites significant different between IDH group and SDHs were annotated to KEGG pathways, and the enrichment of metabolite pathways were shown. **E-F,** The significant matched KEGG pathway terms based on altered metabolites between patients with IDH and ISH were described in the plots. The dots were colored with the -lnP value, and where the color was darker, the enrichment degree was more significant. The dots were further sized according to value of impact factor in each pathway.


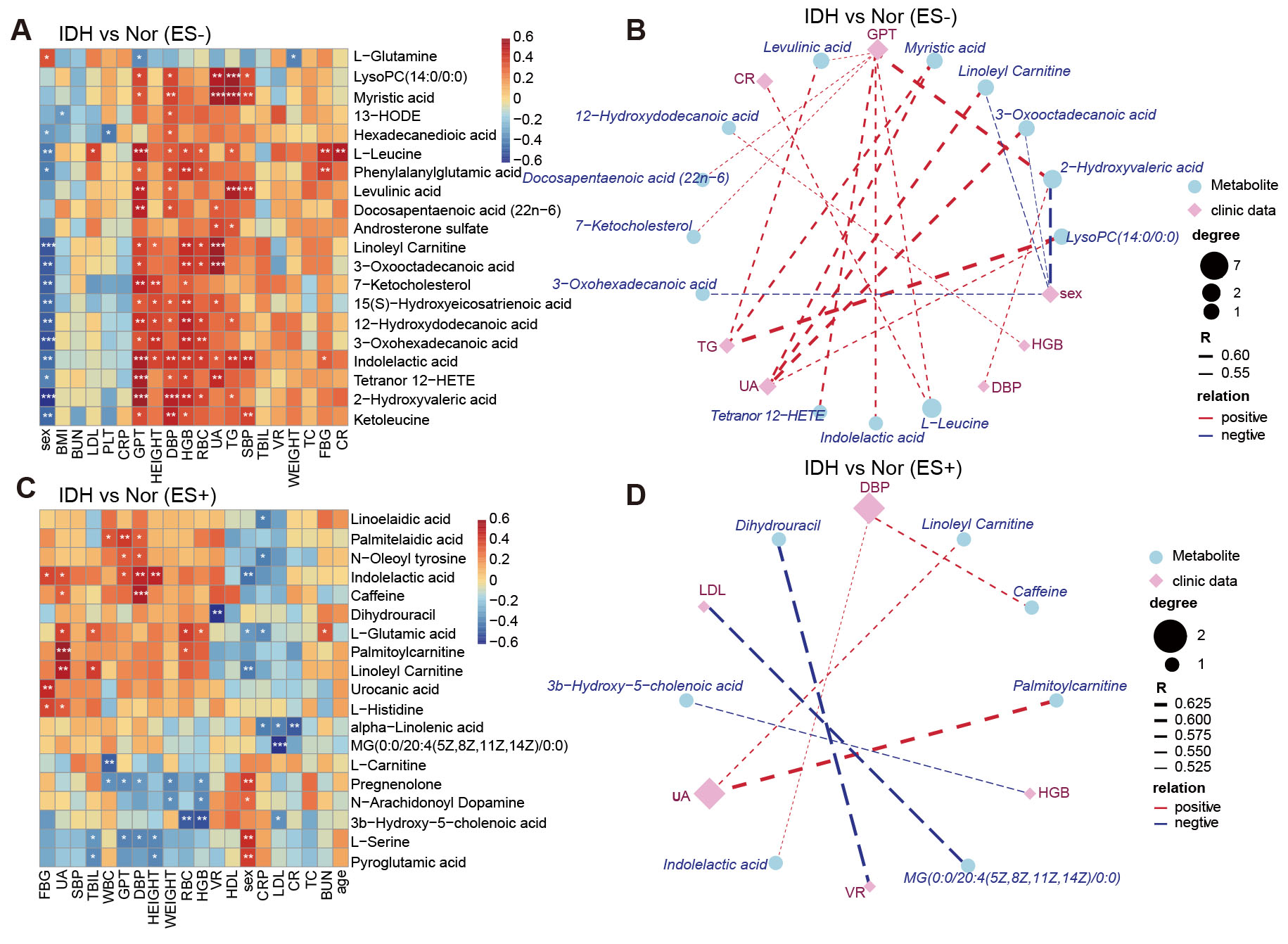
**Figure S5. Serum metabolites differentiating IDH vs. control individuals were associated with gut microbial genera and species. A, C, E, G,** Heat-map showing the correlation of gut genera and species with serum metabolites specific to IDH in ES- and ES+. The genera, species and metabolites included in the analysis were those significantly different between IDH and Nor individuals. A and C were correlation of top 30 metabolites, with genera and species in ES-, E and G were top20 metabolites in ES+. Red indicated positive association, and blue denoted negative association. *p < 0.05, **p < 0.01, and ***p < 0.001, from Spearman’s rank correlation. **B, D, F, H,** Correlation network was obtained by integration of microbiome and metabolome. Blur circles denoted metabolites, and purple rhombus represented microbiota. Circles or rhombus size represented degree. The cutoff for correlation coefficient was ≥0.5 or ≤−0.5, and p value was <0.05 from Spearman correlation. The thickness of the lines was consistent with the correlation coefficient.


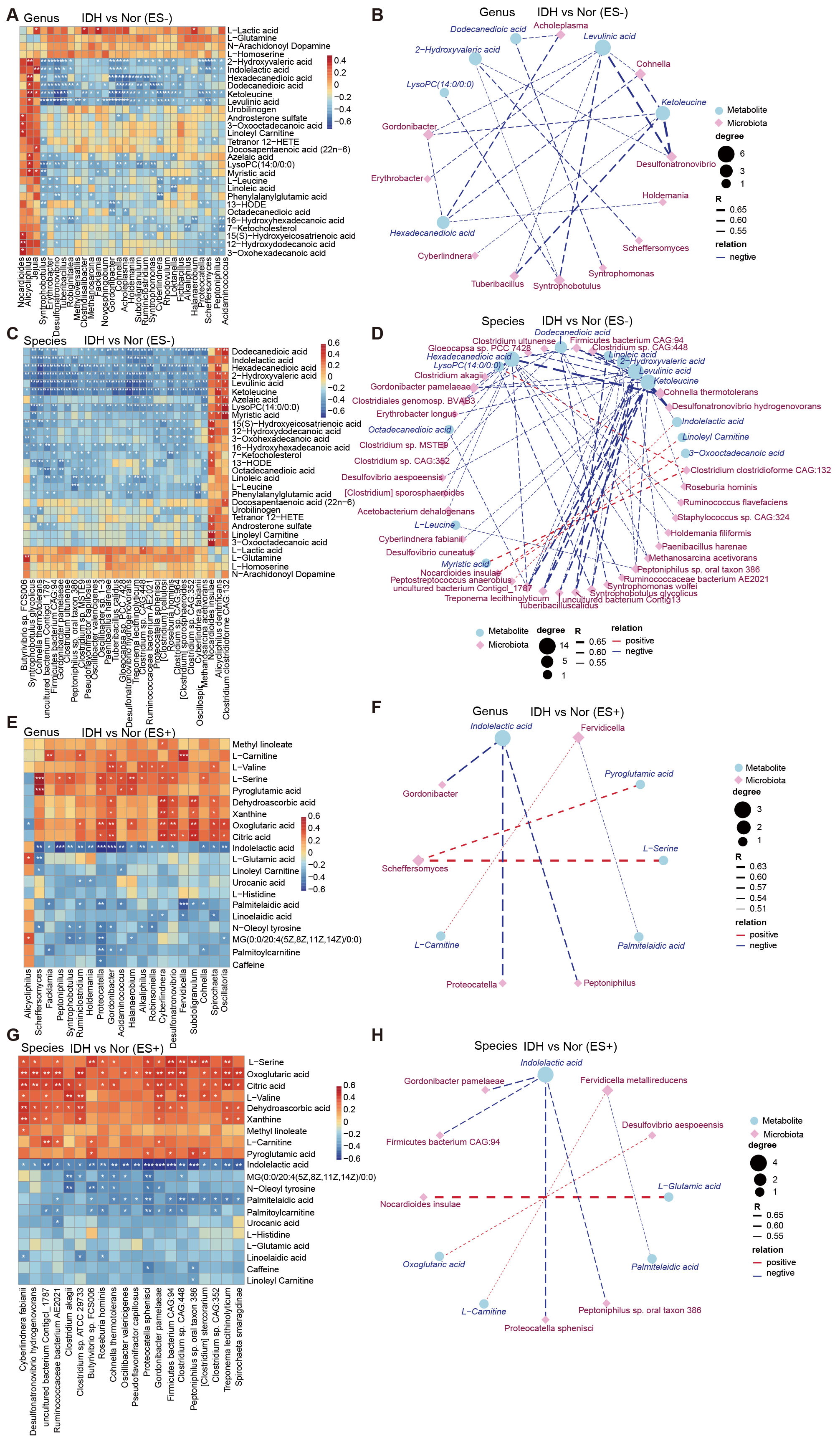
**Figure S6. Profound association between serum metabolites and gut microbiota differentiating SDH patients and controls. A, C, E, G,** Potential relationship of top25 serum metabolites and gut microbial genera/species with discrimination between SDH and Nor was shown. The genera and species were those significantly discrepancy between SDH vs. Nor. The results at genus and species level were represented in A/E and C/G, respectively. Positive correlations were colored in red, and negative associations were represented in blue. *p < 0.05, **p < 0.01, and ***p < 0.001, Spearman’s rank correlation. **B, D, F, H,** Network describing the linkage between serum metabolome and fecal microbiome. Metabolites were shown with purple circles, and gut microbiota were with blue rhombus. Size of circles or rhombus denoted the degree. The threshold of correlation coefficient was |value| ≥0.3, and p <0.05 (Spearman correlation). The correlation coefficient was exhibited with the thickness of the connecting line, and the positive or negative correlation between nodes was indicated with red or blue color.


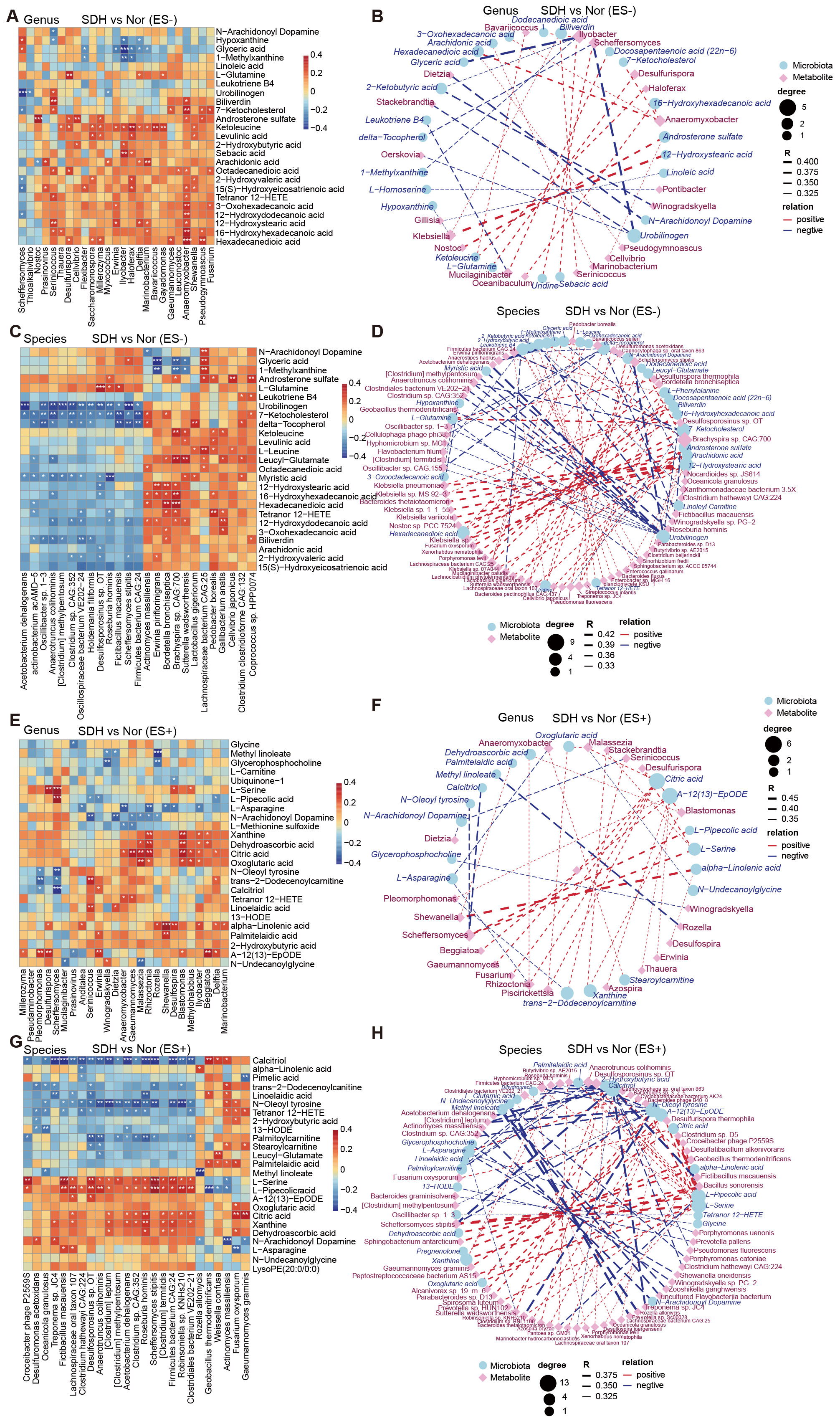
**Figure S7. The association of clinical parameters with the serum metabolites varied in IDH. A, C,** Heat-map depicting Spearman’s rank correlation of the top20 differential metabolic compounds between groups and the clinical indexes of individuals. A showed the differently abundant metabolites between IDH and control in ES- were related with clinical characteristics. C was the relationship of distinct metabolites in ES+ when comparing IDH and controls, with demographic characteristics of the subjects. Red indicated positive association; blue represented negative association. The statistical significance was expressed with *p < 0.05, **p < 0.01, and ***p < 0.001, respectively. **B, D,** Correlation network depicting the prominent linkage between various metabolites and clinical parameters of the subjects. The correlation coefficient was ≥0.5 or ≤−0.5, and p values were <0.05, derived from Spearman correlation analysis. Blue circles denoted metabolites, and purple rhombus represented clinical parameters. Circle or rhombus size represented degree. Red dash lines were positive correlation, while blue lines indicated negative association. Lines thickness was based on the correlation coefficient.


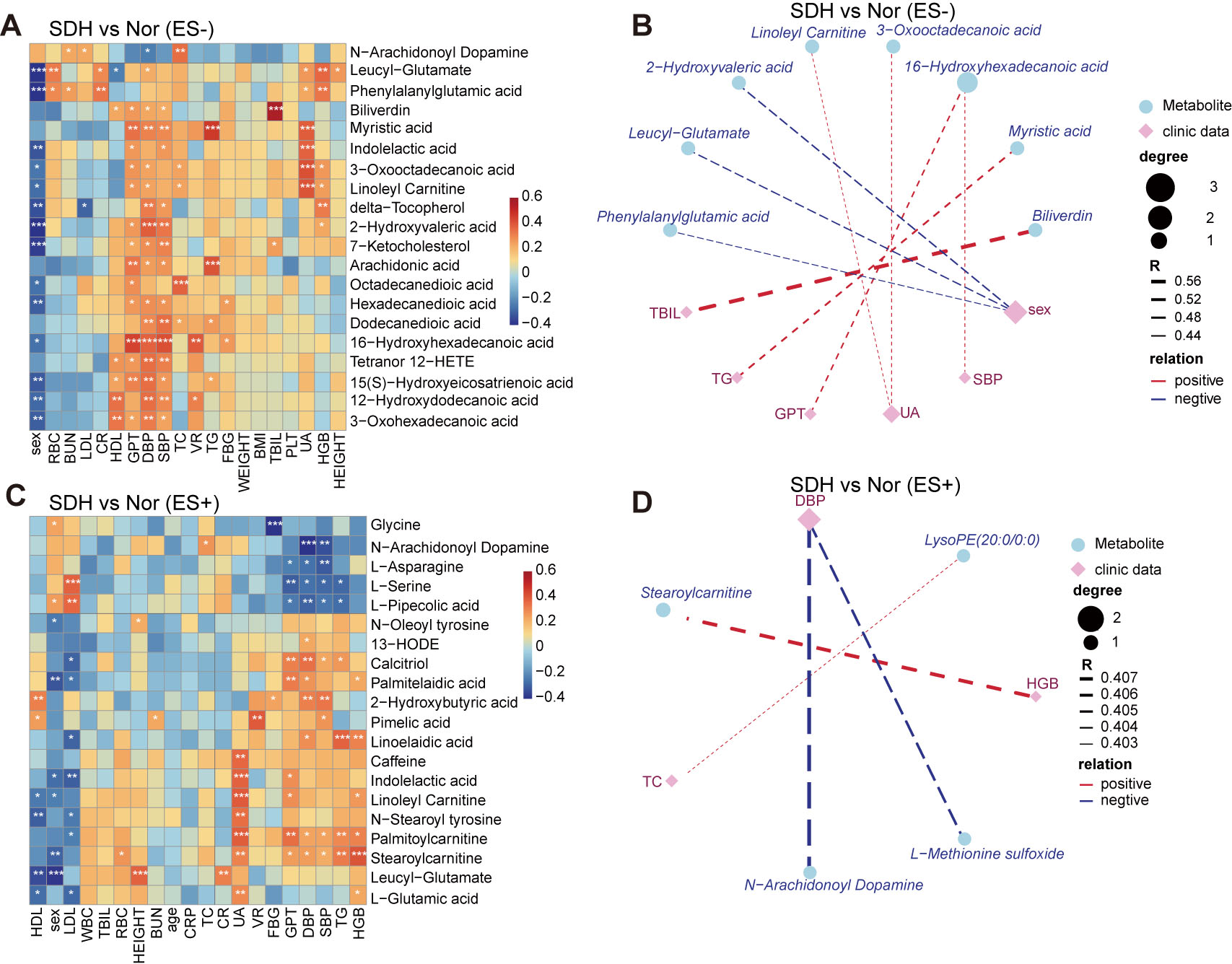
**Figure S8. The association of clinical indexes with the serum metabolites varied specifically in SDH. A, C,** Top20 differently abundant metabolites between SDH and control were related with demographic characteristics of the subjects. Red indicated positive association; blue represented negative association. The statistical significance was expressed with *p < 0.05, **p < 0.01, and ***p < 0.001, respectively. **B, D,** Correlation network illustrating the prominent linkage between various metabolites and clinical parameters. The correlation coefficient was ≥0.4 or ≤−0.4, and p values were <0.05, derived from Spearman correlation analysis. Blue circles denoted metabolites, and purple rhombus represented clinical parameters. Circle or rhombus size represented degree. Red lines were positive correlation, while blue dash lines indicated negative association. Lines thickness was based on the correlation coefficient.
